# The investment case as a mechanism for addressing the NCD burden: Evaluating the NCD institutional context in Jamaica, and the return on investment of select interventions

**DOI:** 10.1371/journal.pone.0223412

**Published:** 2019-10-04

**Authors:** Brian Hutchinson, Roy Small, Kofi Acquah, Rosa Sandoval, Rachel Nugent, Tamu Davidson, Delia Itziar Belausteguigoitia, Nicholas Banatvala, Douglas Webb, Dudley Tarlton, Alexey Kulikov, Elisa Prieto, Karin Santi

**Affiliations:** 1 Center for Noncommunicable Diseases, RTI International, Seattle, Washington, United States of America; 2 HIV, Health and Development Group, United Nations Development Programme, New York, New York, United States of America; 3 Social Policy, Health and Economics Research (SPHERE), RTI International, Research Triangle Park, North Carolina, United States of America; 4 Noncommunicable Diseases and Mental Health, Pan American Health Organization, Washington, D.C., United States of America; 5 Noncommunicable Diseases and Injuries Prevention, Jamaica Ministry of Health, Kingston, Jamaica; 6 UN Interagency Task Force on the Prevention and Control of Noncommunicable Diseases, World Health Organization, United Nations, Geneva, Switzerland; 7 Bureau Policy and Programme Support, United Nations Development Programme, New York, New York, United States of America; 8 Noncommunicable Diseases and Mental Health, Caribbean Subregional Program Coordination, Pan American Health Organization, Bridgetown, Barbados; 9 HIV, Health and Development Group, United Nations Development Programme, Panama City, Panama; Centers for Disease Control and Prevention, UNITED STATES

## Abstract

Noncommunicable diseases (NCDs) are a broad challenge for decision-makers. NCDs account for seven out of every 10 deaths globally, with 42 percent occurring prematurely in individuals under age 70. Despite their heavy toll, NCDs are underfunded, with only around two percent of global funding dedicated to the disease set. Country governments are responsible for funding targeted actions to reduce the NCD burden, but among other priorities, many have yet to invest in the health-system interventions and policy measures that can reduce the burden. This article examines “investment cases” as a potential mechanism for catalyzing attention to—and funding for—NCDs. In Jamaica, using the UN inter-agency OneHealth Tool, we conducted an economic analysis to estimate the return-on-investment from scaling up strategic clinical interventions, and from implementing or intensifying policy measures that target NCD risk factors. In addition, we conducted an institutional and context (ICA) analysis, interviewing stakeholders across sectors to take stock of promising policy pathways (*e*.*g*., areas of general consensus, political appetite and opportunity) as well as challenges to implementation. The economic analysis found that scaling up clinical interventions that target CVD, diabetes, and mental health disorders, and policy measures that target tobacco and alcohol use, would save over 6,600 lives between 2017–2032, and avert JMD 81.3 billion (USD 640 million) in direct and indirect economic costs that result from mortality and morbidity linked to NCDs. The ICA uncovered government economic growth targets and social priorities that would be aided by increased attention to NCDs, and it linked these targets and priorities to the economic analysis.

## Introduction

Encompassing a wide range of diseases and mental and substance use disorders, non-communicable diseases (NCDs) account for seven out of every 10 deaths globally, with 42 percent of all NCD deaths occurring prematurely (among individuals under the age of 70) [1]. Moreover, the NCD burden is driven by a complex array of factors, including increasing life spans, and changes in urbanization, trade, and globally integrated markets that have increased populations’ exposure to environmental and behavioral risk factors [2] (*e*.*g*., air pollution, chemicals, tobacco and alcohol use, unhealthy diets, and physical inactivity).

Cost-effective interventions to address NCD risk factors exist. However, many of these interventions require the meaningful engagement of sectors beyond health (*e*.*g*., ministry of finance collaboration to increase tobacco or alcohol taxes). Achieving multisectoral NCD action can be complex, especially when commercial and public-private interests linked to NCD risk factors lead to real or perceived incentive conflicts on whether to implement—and how to structure—policy measures. An additional challenge is oft competing perspectives, amongst both decision-makers and the general public, on the role of the individual versus the state in addressing the problem, particularly in relation to behavioral risk factors.

As part of broader efforts to accelerate progress on SDG Target 3.4 to reduce premature mortality from NCDs by one-third by 2030, the World Health Organization (WHO) and United Nations Development Programme (UNDP)—in partnership with ministries of health—began conducting a series of national investment cases that examine the costs of NCDs, not only to human health, but also to health systems and national economies. The investment case initiative arose from national authorities’ expressed interest in economic arguments that can be used to advocate for NCD action to ministries of finance and other economic sectors of government, and support to better understand the broader national context in which NCD decisions get made (or not).

The investment cases consist of two components: an *economic analysis* and an *institutional and context analysis (ICA)*. The economic component analyzes the expected return on investment (ROI) from implementing clinical interventions and policies identified as priorities within national strategic plans on NCDs and/or the broader WHO Global NCD Action Plan. The ICA, through key informant interviews, aims to uncover the most promising policy pathways (*e*.*g*., areas of general consensus, political appetite and opportunity) as well as challenges to implementation.

This article discusses the “investment case” as a tool to catalyze national-level action on SDG Target 3.4. The article outlines the methodology behind the economic analysis and ICA, and presents results from an investment case conducted in Jamaica in 2017. The final section summarizes the Jamaica experience and future development of NCD investment cases.

### Why an investment case for NCDs?

Despite the fact that NCDs account for 60 percent of disability adjusted life years globally [3], less than two percent of global donor funding on health is allocated to NCD prevention and control [4]. With only small average growth (1.8%) in overall donor assistance for health from 2010–2016 [4] and a growing shift toward domestic responsibility for financing health and development [5], low- and middle-income countries are reliant on national resources to reduce premature mortality from NCDs by one-third by 2030, in line with SDG Target 3.4. In turn, Target 3.4 is situated amongst 168 other SDG targets, all ostensibly competing for limited domestic resources. Thus, evidence that clarifies investment decisions can help governments allocate priorities and better understand their interactions.

In addition, many behavioral risks present a unique challenge in regard to their connection to the economy. Certain products which exacerbate NCD burdens (e.g., tobacco, alcohol, and processed foods and beverages high in sodium, trans fatty acid and/or sugar) are often strongly integrated in national market economies. Such products are deeply tied to industry and trade, and are bases of government revenue (e.g., through taxation). Thus, there is a need to provide evidence that includes both the public health argument—given that health authorities and public health concerns are often not represented—*and* the economic rationale for intervention. Without both, decision makers have incomplete information on *why* to act, presenting a barrier to NCD action, particularly amongst the economic sectors needed to finance and implement key interventions. Decision makers also need country-specific evidence on *how* to act, including the cost-effectiveness of interventions and policy measures in order to efficiently allocate scarce resources.

## The investment case for the prevention and control of NCDs in Jamaica

In Jamaica, nearly four out of every five individuals dies from an NCD-related cause, with 31 percent of all NCD deaths occurring before the age of 70 [1]. Beyond the toll on human health, NCDs also impose an economic burden on households, health systems, and national economies. In 2012, Jamaica spent about 15 percent of its health budget on the four main NCDs alone (CVD, diabetes, cancer, and COPD) [6, 7].

These high expenditures impose a *direct* economic burden on the country, but the economic burden of NCDs also stems from *indirect* sources. Poor health reduces output by permanently or temporarily removing individuals from formal or informal labor markets. When individuals die prematurely, the labor output that they would have produced in their remaining years is lost. In addition, individuals with NCDs are more likely to *not* participate in the workforce [8], to miss days of work (absenteeism) and/or to work at a reduced capacity while at work (presenteeism) [9, 10].

### Materials and methods

#### Economic analysis

To analyze the extent to which the NCD burden can be reduced, the investment case follows six methodological steps: 1) Select policy measures and interventions for analysis; 2) Assess the baseline coverages of each policy measure and intervention, and the target goals for scale-up over the 15-year period; 3) Estimate the health gains that can be achieved as a result of implementation and scale up; 4) Estimate the financial costs to achieve those health gains; 5) Monetize health gains to assess the impact on the labor force and economic output, and: 6) Calculate the net benefits and return on investment of each policy measure and intervention. The *S1 Appendix* provides details on each methodical step, describing the clinical interventions and policies that were selected for analysis; their baseline and target level of implementation; the expected impact of the interventions (effect sizes), and their financial costs. In addition, the appendix provides details on the steps used to monetize health gains.

Following priorities outlined by the Jamaica Ministry of Health (MoH), the investment case assesses clinical interventions that target NCDs (cardiovascular disease, diabetes) and mental health disorders (depression, anxiety), and policy measures that address NCD risk factors (tobacco, alcohol). National surveys, national plans, regulations, academic literature, WHO databases, and opinions from MoH officials were used to assess baseline coverage rates of clinical interventions and what policy measures Jamaica currently has in place. MoH officials provided target goals for implementing new policies—or intensifying existing ones—and scaling up clinical-intervention coverages over a 15-year period simulating the length of the SDG era (see *S1 Appendix*).

Over the 15-year period, the health benefits that result from moving policies and clinical interventions from baseline to target goals are obtained using the NCD Impact Module of the inter-UN agency OneHealth Tool (OHT). The impact module contains a collection of multistate lifetables that model the extent to which the population experiences health events and the likelihood of death (see *S1 Appendix*). The Tool has been used in other published return on investment studies [11–13].

Health gains are monetized to represent the economic value of improvements in health. The economic value is a function of the additional amount of time that an individual can spend engaged in productive economic activities as a result of being in a state of good health, where additional time is valued using the human capital approach (see S1 Appendix). The value of avoided premature mortality is obtained by multiplying the number of deaths avoided by labor force participation rates and the expected economic contribution of each worker (valued at GDP per worker). Estimates of the extent to which better health decreases labor force exit, absenteeism, and presenteeism are obtained from the literature, with restored productive time due to interventions valued at GDP per worker.

The financial costs to the government of implementing tobacco and alcohol policy measures—or of intensifying or enforcing existing ones—are estimated using the Excel-based WHO NCD Costing Tool. Clinical-intervention costs are calculated using an ingredients-based approach, where the quantity of resources used in an intervention was multiplied by the resource’s unit cost to obtain the average cost of providing one individual with treatment. Treatment assumptions are drawn from the OneHealth Tool, and unit costs of individual resources are sourced from local government and non-government sources in Jamaica. The total cost of providing services is obtained by multiplying the unit cost of providing a service by the number of additional services provided in the intervention scenario.

The return on investment (ROI) is calculated for groups of interventions, or “packages”, that target specific diseases, disorders, or risk factors (e.g., CVD, depression, tobacco use). For each package, the ROI is calculated by dividing the economic value of the gains from investments by their respective costs. While the costs, benefits, and a ROI are also reported for “all packages” in combination, these totals merely add each packages contribution together. The OHT model does not take into account the overlapping effects that implementing all packages may have in combination.

Future costs and benefits are discounted at a rate of three percent. Jamaican dollars are translated to US dollars using a 127 JMD: 1 USD exchange rate [14].

#### Institutional context analysis

Sampling and recruitment for key informant interviews (Box 1) was carried out in line with the ICA guidance tool developed by UNDP (S2 Appendix), with intent to engage high-level stakeholders across sectors (e.g., politics, government, civil society, media) who were perceived as relevant to NCDs in the Jamaica context. The need for ethical approval of the key informant interviews was waived by the Research Triangle Institute (RTI) Institutional Review Board. Informants’ consent for the interviews was obtained via written correspondence between the UN and the Government of Jamaica. Interviews with key stakeholders were semi-structured, focusing on a framework of questions (S2 Appendix) that assessed the political dimensions of NCD policy adoption, implementation, and enforcement.

**Box 1. Stakeholders consulted during the Jamaica ICA (March 27–31, 2017).**

- Ministry of Health (MoH): Health Promotion and Protection Branch, Health Services Planning and Integration Branch, Health System Improvement Branch of the Health Policy Planning and Development Division, National Epidemiology Unit, Standards and Regulation.
- Ministry of Health Agencies: National Health Fund (NHF), National Council on Drug Abuse (NCDA) and National Public Health Laboratory
- Ministry of Finance and Public Service (MoF)
- Ministry of Industry, Commerce, Agriculture and Fisheries (MICAF)
- Ministry of Economic Growth and Job Creation (MEGJC)
- Ministry of Education, Youth and Information (MoE)
- Ministry of Labour and Social Security (MLSS)
- Ministry of Foreign Affairs and Foreign Trade (MFAFT)
- Ministry of Transport and Mining (MTW)
- Ministry of Culture, Gender, Entertainment and Sports (MCGES)
- Ministry of Local Government and Community Development (MLGCD)
- Jamaica Chamber of Commerce
- Civil society representatives from the Heart Foundation of Jamaica, Diabetes Association of Jamaica Cancer Society and National Consumers League.
- Local media representatives from the Gleaner company.

### Results–economic analysis

The OneHealth Tool models the incidence of disease averted, healthy life years gained, and the number of lives that are saved. Table 1 describes the health benefits generated by each package of interventions. The package of CVD interventions provides the largest mortality impact, saving 4,358 lives over 15 years. Lives saved are mediated through avoided CVD events: strokes and myocardial infarctions. Epidemiological modelling in the OHT suggests that 75,858 strokes and 62,500 IHD events will occur in Jamaica from 2017–2032. Over that time, the CVD package reduces the number of strokes by 6,068 (7.9%), and the number of IHD events by 4,346 (6.9%).

**Table 1:** Estimated health benefits over a 15-year time horizon, by intervention package.

Intervention package^a^	Strokes averted	IHD events averted	Cases of blindness averted	Cases of lower-limb amputation averted	Remission from episodes of depression or anxiety	Mortality averted	Healthy life years gained
CVD	6,068	4,346	--		--	4,358	30,456
Tobacco	1,176	967	--		--	597	7,355
Alcohol	--	--	--		--	518	23,292
Diabetes	--	--	3,106	836	--	262	4,419
Depression	--	--	--		120,259	911	51,328
Anxiety	--	--	--		108,968	--	22,671

^*a*^ Interventions and policy measures that target the same disease, risk factor, or mental health condition are bundled together as “packages” for analysis (see S1 Appendix). For example, the interventions addressing diabetes or diabetes complications (i.e. glycemic control, screening and treatment of retinopathy and neuropathy) form the “Diabetes package”.

**Fig 1:**
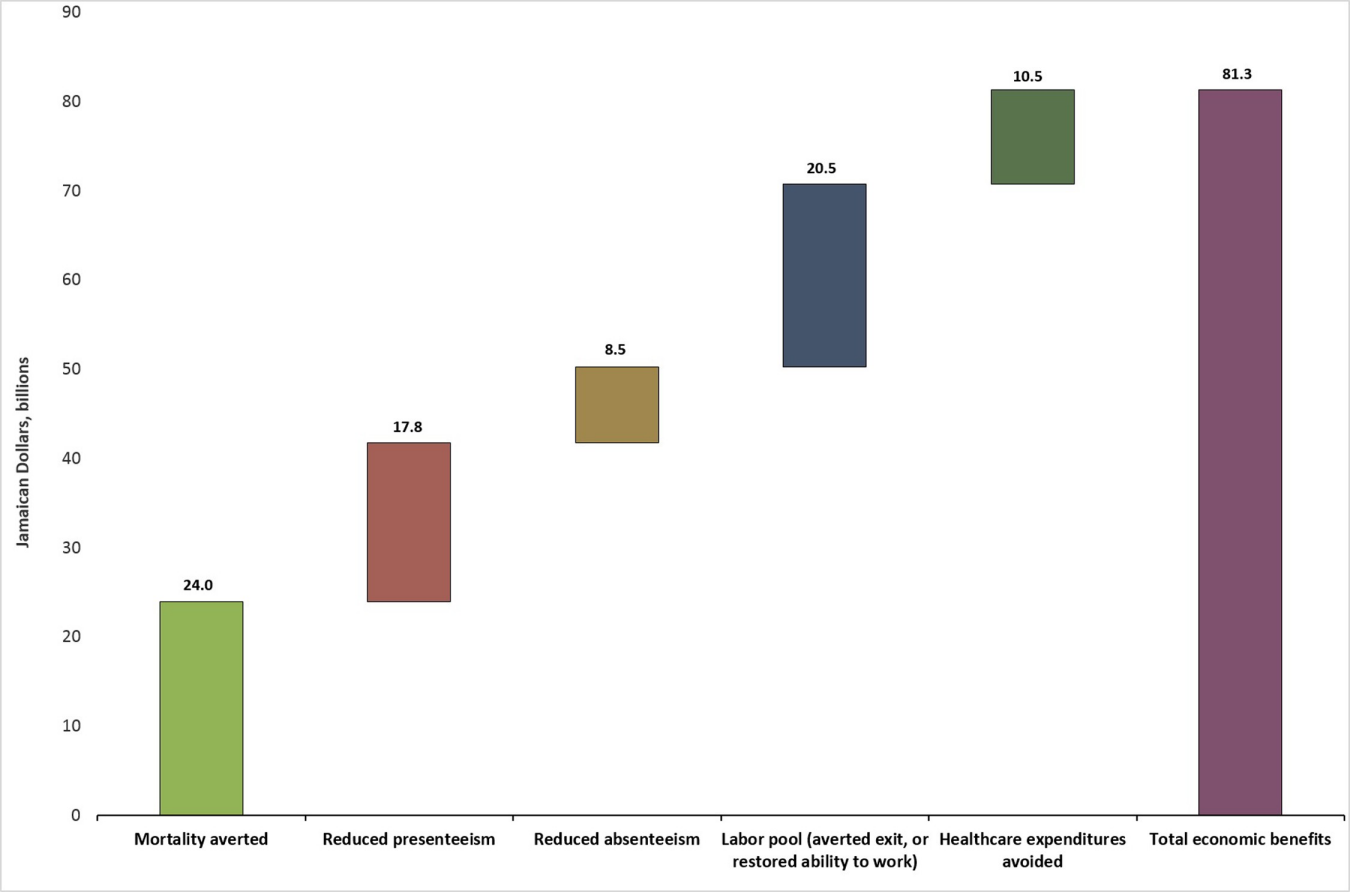
Economic savings from implementing tobacco and alcohol policies, and scaling up clinical interventions to treat CVD, diabetes, depression, and anxiety, 2017–2032 (2016 JMD, billions).

Fig 1 translates the health benefits in Table 1 into the economic savings that can be expected from avoiding direct treatment costs and increasing workforce output.

In total, over 15 years, Jamaica would save JMD 81.3 billion (USD 640 million) that would otherwise be lost if it does not scale up the coverage of clinical interventions, and implement or intensify policy measures to reduce tobacco and alcohol consumption. On average, that is the equivalent of about JMD 5.4 billion (USD 43 million) in annual avoided economic losses.

The avoided economic losses derive from lowering direct and indirect costs of NCDs. With better health, fewer individuals need to be treated for complications from disease, resulting in direct cost savings to the government. In addition, better health leads to increased worker output. Fewer working-age individuals leave the workforce prematurely due to death or disease. Laborers miss fewer days of work (absenteeism) and are less hindered by health complications while at work (presenteeism).

About 87 percent of the savings (JMD 70.7 billion, USD 557 million) are from indirect sources—mortality, presenteeism, absenteeism, labor force exit—while 13 percent of savings (JMD 10.5 billion, USD 83 million) are derived from averted direct healthcare expenditures. The largest gains result from avoided mortality, representing about 29.5 percent of the packages’ total economic benefits (JMD 24 billion, USD 189 million). The next largest gains result from avoided labor force exit, followed by presenteeism, avoided healthcare expenditures, and then absenteeism.

The return on investment for NCD intervention packages was evaluated in the short-term (five years), to align with the current political cycle, and medium- term (15 years). Together, over 15 years, the packages are estimated to cost JMD 37.8 billion (USD 297.5 million), or JMD 2.5 billion annually (USD 19.8 million), and to generate JMD 81.3 billion in total economic benefits.

Table 2 list the ROI of each package of interventions. The anxiety interventions have the highest return on investment (3.5), followed by the tobacco package (3.2), depression (3.0), CVD (2.2), alcohol (1.9), and the diabetes interventions (1.2). While the anxiety package has the highest ROI, interventions treating depression and CVD generate the highest total economic benefits.

**Table 2:** Return on investment, by NCD package (2016 JMD, billions).

	5-year period			15-year period		
NCD intervention packages	Total costs	Total economic benefits	ROI	Total costs	Total economic benefits	ROI
Anxiety	0.7	1.1	1.6	4.7	16.4	3.5
Tobacco	0.6	0.2	0.4	1.2	3.7	3.2
Depression	1.1	2.4	2.1	7.3	21.8	3.0
CVD	1.3	1.1	0.8	9.8	21.2	2.2
Alcohol	0.6	0.2	0.4	1.3	2.5	1.9
Diabetes	2.1	1.1	0.5	13.5	15.7	1.2
**All packages**^**a**^	**6.3**	**6.1**	**0.96**	**37.8**	**81.3**	**2.2**

^a^ The total costs and total benefits presented for “all packages” are assumed to be additive. The investment case analysis does not analyze the synergistic effects that implementing all packages may have in combination.

### Discussion

This section considers the economic modelling results in light of Jamaica’s overall institutional context at the time of the investment case, demonstrating the type of advocacy-centered recommendations for the investment case that can help accelerate a stronger, more coherent national NCD response.

#### (1) NCD action must be tied to the “bottom line”, considering the current government’s emphasis on job creation and economic growth

A major objective of the investment case is to support governments to perceive NCD action as an economic opportunity, versus the frequently default perception that such action, especially fiscal and regulatory measures, would harm the economy overall. In 2016, the Prime Minister and Government-established Economic Growth Council declared intentions to grow GDP in Jamaica by an ambitious five percent in four years [15]. These intentions, known short-hand as ‘5 in 4’, would mean growth of JMD 87.9 billion (USD 690.9 million) between 2017 and 2020. This ambition presents an opportunity to re-frame NCD action as a contributor to national prosperity, both during and after the ‘5 in 4’ period.

Investing in the small package of interventions analyzed *would* contribute to economic output. In the first four years after implementation, Jamaica would realize JMD 3.2 billion (USD 26 million) in indirect economic gains as a result of improved health, equivalent to about 3.6 percent of its GDP growth goal. Over the fifteen-year period (2017–2032), implementing the analyzed interventions would help Jamaica to achieve JMD 70.7 billion (USD 557 million) in indirect economic gains.

Despite these benefits, improved health—much less NCD action—is not specified in the government’s ‘5 in 4’ plan as an economic growth contributor. This omission should be stressed in discussions with relevant Ministries, and Jamaica’s Economic Growth Council and its Chamber of Commerce should be supported to see NCD action as vital to increasing member firms’ competitiveness, productivity and efficiency.

#### (2) NCD action must also be tied to Jamaica’s leading social priorities, to boost the economic frame

It is crucial that NCD action be framed as promotive of Jamaica’s social priorities, considering the current government’s emphasis on general well-being (GWB) alongside GDP growth. The strongest social entry point is the government’s goal of providing universal access to health services. This goal has significant political capital, with both the current government and its opposition reportedly united on the issue, and the Prime Minister having deemed universal access to health services “the right thing to do”, suggesting a human rights imperative.

Contributing to universal health access, the investment case package would restore 139,521 healthy life years and unlock JMD 10.5 billion (USD 83 million) in averted medical costs. This would reduce burdens on Jamaica’s already stretched health system (as well on civil society to fill public sector gaps).

Realizing such benefits requires a financial commitment from the government that is equivalent to about a four percent increase in annual government health expenditures. In addition, while the investment case takes into account the cost of human resources to provide a greater number of services, it assumes that the human capital required to provide those services can be acquired. Additional funding beyond that accounted for in the investment case may also be needed to develop health system capacity. Innovative financing mechanisms may be required to fund NCD efforts. Two policy measures, the tobacco and alcohol tax increases, would reduce consumption of health-harming products while generating revenue which could help finance Jamaica’s National Health Fund. Taxation of sugar-sweetened beverages (SSBs), though not modelled in the economic analysis, should also be explored for its health and revenue generating potential [16], considering the success of Mexico’s SSB tax in reducing consumption, especially amongst the poorest [17].

Beyond universal access to health services, other social priorities that NCD action would advance include child protection, reduction of both crime and violence. For example, banning sodas in schools and taxing SSBs, both of which Jamaica is considering, would help protect children from obesity, type 2 diabetes and other NCDs later in life. Reducing harmful use of alcohol would help address violence (a major source of public [18] and economic [19] concern).

#### (3) To maximize the investment case opportunity, existing high-level leadership to address NCDs must be leveraged, multisectoral governance for NCDs strengthened, and better policy coherence achieved

NCD action requires strong leadership and multisectoral coordination. The Prime Minister of Jamaica has stressed the need for NCD action, and the Health Minister is a public champion. These and other influential actors, such as the Minister of Finance, should be supported to advocate investment case recommendations to all stakeholders as a *national development* opportunity, not just a *health* opportunity. The Ministry of Health has built a solid foundation for stronger whole-of-government NCD action and greater public awareness. It has cultivated strong relationships with relevant ‘non-health’ ministries, and led the development of several multisectoral plans, policies and programs on NCDs. It has also spearheaded health promotion campaigns, a prominent example being *Jamaica Moves*, a national campaign centered on physical activity that is being broadened to all Caribbean countries. With the National Strategic and Action Plan for the Prevention and Control of NCDs in Jamaica 2013–2018 ended, the next iteration could include the investment case recommendations (e.g., higher intensity tobacco and alcohol control legislation). Other sectors could also include NCD investment case results in *their* new and revised plans, which would further position NCDs as a sustainable development issue.

#### (4) Shift NCD framings away from being an issue of “personal responsibility” or a “byproduct of culture”

Many stakeholders described NCDs mostly as an issue of personal culpability or of inadequate willpower. These stakeholders tended to stress “lifestyle”, “choice”, and not wanting to become a “punitive society”; they emphasized education and awareness-based approaches. Such frames do not capture the fact that NCD burdens are rooted in social inequities, intensified by commercial factors, and mutable through decisive government action and leadership. Encouragingly, some stakeholders recognized the responsibility of government to foster enabling environments and contexts that make the healthy choice easier and more attractive for citizens. These stakeholders frequently discussed notions of “support”, “fairness”, “protection” and “empowerment.”

Given Jamaica’s lift-yourself-up national spirit and resilience, it would benefit from health promotion efforts which support positive individual behavior change. A major opportunity is to broaden the successful physical activity campaign, *Jamaica Moves*, so that it ultimately represents progress on *all* aspects of the national NCD response.

Stakeholders also diverged on the role of culture. Some stakeholders pinned the NCD epidemic almost entirely on culture, noting that Jamaica is a sugar-producing nation that loves its rum, fried food and “fluffy women.” Others acknowledged that Jamaican culture extends back centuries, but that the NCD epidemic is relatively recent. They noted a dissonance between traditional culture, for example Jamaica’s Rastafarian roots encouraging healthy eating, and the current influx of fast, processed foods including high fructose corn syrup. Virtually all stakeholders agreed that certain industries (e.g., tobacco, alcohol, food) are worsening the problem, largely through targeted marketing campaigns.

Neither government nor civil society has the financial resources to compete with these industries’ efforts, but they do have the political and advocacy power to more strongly regulate clear threats to health and well-being.

## Conclusion: Reflections and takeaways from the experience

Findings from the investment case have been presented at several local, regional and global meetings, including: the annual Ministry of Health conference on NCDs; a symposium organized by the Jamaica University of Technology on the challenge of obesity and NCDs in the Caribbean, and; at the April 2018 WHO Global dialogue on financing for prevention and control of NCDs.

The investment case has also been used as evidence to support policy. The Minister of Health emphasized the investment case findings to support health priorities in a speech to the House of Representatives in June 2018, and the report is being referenced as supporting evidence for Ministry of Health program priorities and plans. The Minister of Finance has stressed the results in presentations in order to call for changes and more investment in health.

The Ministry of Health reports that findings have resonated with stakeholders, and filled an evidence gap by making locally relevant arguments for investing in NCDs. In addition, the investment case process brought multiple sectors together around the common theme of NCDs, forming a platform for future multi-sectoral responses.

Use of results to advocate for increased funding and targeted action indicates some promise for investment cases to act as a mechanism for spurring change that can reduce the NCD burden. Demonstrating demand for this kind of evidence, WHO and UNDP have received requests for NCD investment cases from nearly 50 countries. The Framework Convention on Tobacco Control Secretariat, UNDP and WHO are also conducting 15 national “tobacco control” investment cases specific to WHO FCTC implementation. At its best, well-timed, locally relevant evidence can prompt governments to increase affordable access to clinical interventions for NCDs while enacting bold fiscal, regulatory and policy measures, in line with Member-State endorsed guidance, to protect populations from NCD risk factors. But, even if these gold standard results are not attained, investment cases can still play a role in raising public and political awareness around NCDs, better understanding the complex landscape of stakeholders including possibilities for stronger coordination and cooperation, and evidencing cost-effective options to reduce the health and economic burden of NCDs.

## Supporting information

S1 Supporting Appendix Economic analysis–Methods and inputs. (DOCX)

S2 Supporting Appendix Institutional and context analysis—Methods and background. (DOCX)

S1 File OneHealth Tool version 4.45_analysis file. (NC)

S1 Fig Health-state transitions within the OneHealth Tool (OHT) Impact Module's CVD platform. (TIF)

S2 Fig How do diverse institutions in a society shape the likelihood of programme/policy success? (TIF)
