# Correction: The investment case as a mechanism for addressing the NCD burden: Evaluating the NCD institutional context in Jamaica, and the return on investment of select interventions

**DOI:** 10.1371/journal.pone.0230270

**Published:** 2020-03-04

**Authors:** Brian Hutchinson, Roy Small, Kofi Acquah, Rosa Sandoval, Rachel Nugent, Tamu Davidson, Delia Itziar Belausteguigoitia, Nicholas Banatvala, Douglas Webb, Dudley Tarlton, Alexey Kulikov, Elisa Prieto, Karin Santi

Tamu Davidson should be included in the author byline. Tamu Davidson should be listed as the sixth author and affiliated with: Noncommunicable Diseases and Injuries Prevention, Jamaica Ministry of Health, Kingston, Jamaica. The contributions of this author are as follows: Investigation, Project Administration, Writing—original draft, Writing–review & editing.

The correct citation is: Hutchinson B, Small R, Acquah K, Sandoval R, Nugent R, Davidson T, et al. (2019) The investment case as a mechanism for addressing the NCD burden: Evaluating the NCD institutional context in Jamaica, and the return on investment of select interventions. PLoS ONE 14(10): e0223412. https://doi.org/10.1371/journal.pone.0223412

## References

[1] Hutchinson B, Small R, Acquah K, Sandoval R, Nugent R, Belausteguigoitia DI, et al (2019) The investment case as a mechanism for addressing the NCD burden: Evaluating the NCD institutional context in Jamaica, and the return on investment of select interventions. PLoS ONE 14(10): e0223412 10.1371/journal.pone.0223412 31584979 PMC6777795

