# Correction: The investment case as a mechanism for addressing the NCD burden: Evaluating the NCD institutional context in Jamaica, and the return on investment of select interventions

**DOI:** 10.1371/journal.pone.0233140

**Published:** 2020-05-07

**Authors:** The PLOS ONE Staff

## Notice of republication

This article [1] was republished on April 29, 2020, to correct errors in the Funding statement. The publisher apologizes for these errors. A correction [2] to this article was published on March 4, 2020, to address errors in the author byline and citation. The updates outlined in [2] have been incorporated into the republished article. Please download this article again to view the correct version.
