# Supplementary material for: The investment case as a mechanism for addressing the NCD burden: Evaluating the NCD institutional context in Jamaica, and the return on investment of select interventions
Source: PLoS One. 2019 Oct 4;14(10):e0223412. doi: 10.1371/journal.pone.0223412 (PMC6777795; doi:10.1371/journal.pone.0223412)
Supplement: S1 Supporting Appendix — (DOCX) [file pone.0223412.s001.docx]

The Investment Case for Non-Communicable Disease Prevention and Control in Jamaica

S1 Supporting Appendix. Economic Analysis – Methods and Inputs

**Table of contents**

[1. Baseline and target coverages of interventions and policy measures, and scale-up 3](#_Toc16073982)

[2. Health benefits 7](#_Toc16073983)

[2.1 Modeling 7](#_Toc16073984)

[2.2 The impact of clinical interventions and policies that target NCDs 8](#_Toc16073985)

[2.2.1 Clinical interventions 8](#_Toc16073986)

[2.2.2 Policy measures 9](#_Toc16073987)

[2.2.3 Baseline risk factors prevalence 9](#_Toc16073988)

[3. The costs of policy measures and clinical interventions 10](#_Toc16073989)

[3.1 Policy measures 10](#_Toc16073990)

[3.2 Clinical Interventions 10](#_Toc16073991)

[4. Monetizing health benefits: formulas and approach 12](#_Toc16073992)

[4.1 Monetizing gains from avoided mortality 12](#_Toc16073993)

[4.2 Monetizing gains from increases in the labor force 12](#_Toc16073994)

[4.3 Monetizing gains in labor productivity 12](#_Toc16073995)

[4.4 Estimating the value of avoided healthcare expenditures 13](#_Toc16073996)

[4.5 The Return on Investment 13](#_Toc16073997)

[5. References 14](#_Toc16073998)

Introduction to Supplementary Appendix 1

The investment case economic analysis examines the health and economic benefits of implementing policy measures that address NCD risk factors, and of scaling up clinical interventions to prevent and treat NCDs and mental health disorders. The time horizon of the analysis is 2017-2032.

The major methodological steps of the economic analysis are to: 1) **Select policy measures and interventions** for analysis; 2) **Assess the baseline coverages** of each policy measure and intervention, and the target goals for scale-up over the 15-year period; 3) **Estimate the health gains** that can be achieved as a result of implementation and scale up; 4) **Estimate the financial costs** to achieve those health gains; 5) **Monetize** **the health gains** to assess the impact on the labor force and on economic output, and: 6) **Calculate the return on investment** of each policy measure and intervention.

Supplementary Appendix 1 provides additional information on methods, data, and inputs used to perform these steps.

# Baseline and target coverages of interventions and policy measures, and scale up^a^

S1 Table 1: Policies and interventions included in the investment case analysis – Current state of implementation in Jamaica and target goal analyzed in the investment case

| **Intervention** | **State of implementation in Jamaica (2017 baseline)** | **Investment case 15-year target goal** | **Source of baseline coverage  (with brief justification)** | **Source of target goal**  **(With brief justification)** |
| --- | --- | --- | --- | --- |
| **Policy Measures** | | | | |
| **Tobacco** | | | | |
| Enforce bans on smoking in all public places | Smoking is banned in all public places, but is not well enforced in restaurants, cafes, bars, and pubs. | Smoking is banned in all public places, and restaurants, cafes, bars, and pubs are compliant. | WHO Report on the Global Tobacco Epidemic: Country profile – Jamaica [1] | In alignment with Article 8 of the Framework Convention for Tobacco Control (FCTC), the target goal is full compliance with the current law banning smoking in public places—meaning 100 percent of public places are smoke free *and* compliant with the existing law. |
| Institute mass media campaigns | No tobacco mass media campaign has been run since 2013/2014. | Institute a sustained national mass media campaign that airs on TV and radio, and that includes all-other WHO-recommended characteristics (see target column at right). | Conversations with the Jamaican Ministry of Health and the WHO Report on the Global Tobacco Epidemic: Country profile – Jamaica | Per recommendations in the 2017 WHO Tobacco report [2], the target goal is to run a mass media campaign w/ all recommended characteristics, meaning it is part of a comprehensive tobacco control program; is researched and tested with a target audience; includes a media planning and buying process; airs on radio/TV; achieves coverage with journalists, and; has an evaluation component. |
| Implement and enforce bans on tobacco advertising, promotion, and sponsorship (APS) | Tobacco advertising is banned on TV & radio, but other forms of advertising, promotion, and sponsorship are permitted. | Ban all forms of advertising, promotion, and sponsorship. | Conversations with the Jamaican Ministry of Health and the WHO Report on the Global Tobacco Epidemic: Country profile – Jamaica [1] | In alignment with Article 13 of the FCTC, the target goal is to ban all forms of advertising (e.g., TV, radio, billboards, print, internet), promotion (e.g., point-of-sale product displays, promotional discounts), and sponsorship. |
| Increase taxes on tobacco products | The share of taxes as a percentage of the most sold brand of cigarettes is 44%. | Increase taxes in order to raise the average price of cigarettes by 28 percent. | WHO Report on the Global Tobacco Epidemic: Country profile – Jamaica [1] | In alignment with Article 6 of the FCTC, and obligations to increase taxes in order to reduce the affordability of tobacco products. |
| Implement plain/standardized packaging of tobacco products | Plain packaging is not mandated for tobacco products. | Mandate plain packaging of tobacco products. | Conversations with the Jamaican Ministry of Health and the WHO Report on the Global Tobacco Epidemic: Country profile – Jamaica [1] | The target goal is implementation of plain packaging for tobacco products, in line with the guidelines for implementation of Article 11 of the FCTC. |
| **Alcohol** | | | | |
| Restrict the availability of retailed alcohol | No national regulations limit the number or location of alcohol outlets, or the days, hours, or modes of sale | The number and location of alcohol outlets are regulated, as are days/hours/modes of sale. | Conversations with the Jamaican Ministry of Health and a review of alcohol-related regulations, including The Spirit License Act, 2012; Broadcasting and Radio ReDiffusion Act, 2008; The Spirit Control Act; TV/Sound Broadcasting regulations. | Target in alignment with the WHO Global Strategy to Reduce the Harmful Use of Alcohol [3]. |
| Implement and enforce restrictions on alcohol advertising | Advertisements cannot display the consumption of alcoholic beverages, but no other regulations are in place | Regulation of the content and volume of advertising, promotion, and sponsorship. | Conversations with the Jamaican Ministry of Health and a review of alcohol-related regulations, including The Spirit License Act, 2012; Broadcasting and Radio ReDiffusion Act, 2008; The Spirit Control Act; TV/Sound Broadcasting regulations. | Target in alignment with the WHO Global Strategy to Reduce the Harmful Use of Alcohol [3]. |
| Increase taxes on alcoholic beverages (beer, wine, and spirits) | Excise taxes as a share of the price of the most sold brand of:  Beer- 16.0%;  Wine-22.0%;  Spirits-26.0% | Excise taxes as a share of the price of the most sold brand of:  Beer- 48.5%; Wine-44.8% Spirits-45.2% | Estimates of the existing share of taxes as a percent of the average price of the most sold brand of various types of alcoholic beverages were obtained from the Pan American Health Organization. | The investment case target is set to induce a 8.4% reduction in alcohol consumption over 15 years. Price elasticities were obtained from Nelson (2013) [4], and used to calculate the increase in prices (and tax share) needed to generate the reduction in consumption. |
| **Clinical Interventions** | | | | |
| **Cardiovascular Disease** | | | | |
| Annual screening for metabolic risk factors | 50% | 80% | The baseline represents the percent of adults age 40+ who are screened for metabolic risk factors (obtained from conversations with the Jamaica MoH [5]). | The target goal to screen 80% of adults age 40+ is derived from the Jamaica NCD Action Plan [6]. |
| Treatment for individuals with high blood pressure and total CVD risk < 20% | 40% | 63.6% | The baseline represents the percent of individuals with high blood pressure who receive pharmacological treatment (obtained from Table 10.9 of the Jamaica Health and Lifestyle Survey [7, Table 10.9]). | The scale up in treatment rates is based off of the increase in screening coverage, where individuals are diagnosed and assumed to move on to receive treatment based on treatment rates among those who are aware of their condition [7]. |
| Treatment for individuals with high cholesterol and total CVD risk < 20% | 11.2% | 35% | The baseline represents the percent of individuals with high cholesterol who receive pharmacological treatment [7, Table 10.9]. |  |
| Treatment for individuals with CVD risk (≥ 20%) | 55.7% | 79.8.% | The baseline represents the percent of individuals with high CVD risk who receive pharmacological treatment (Conversations with the Jamaican Ministry of Health). |  |
| Treat new cases of acute myocardial infarction (AMI) with aspirin | 80% | 100% | The baseline represents the percent of individuals with AMI who receive aspirin (obtained from conversations with the Jamaica MoH [5]). | Target goal obtained from conversations with the Jamaica MoH [5]). |
| Provide multidrug therapy to treat those with established ischemic heart disease | 62.4% | 82.4% | The baseline represents the percent of individuals who—after experiencing an AMI and surviving, receive pharmacological treatment to prevent occurrence of a second AMI or stroke [8, Table 6.5] | Target goal obtained from conversations with the Jamaica MoH [5]). |
| Provide multidrug therapy to treat those with established cerebrovascular disease | 50.1% | 70.1% | The baseline represents the percent of individuals who—after experiencing a stroke and surviving, receive pharmacological treated to prevent occurrence of a second stroke or AMI [8, Table 6.5] | Target goal obtained from conversations with the Jamaica MoH [5]). |
| **Diabetes** | | | | |
| Standard and intensive glycemic control | 71.5% | 89% | The baseline represents the percent of individuals with diabetes who receive pharmacological treatment [7, Table 10.9]. | Reflective of goals stated within the Jamaica National Strategic and Action Plan for NCDs [6] goal to increase the proportion of diabetics aware of—and controlling—their condition. |
| Screening for retinopathy, and laser photocoagulation to treat sight-threatening retinopathy | 7% | 37% | The baseline represents the percent of individuals with sight-threatening retinopathy who are treated with laser photocoagulation (based on estimates provided by the Diabetes Association of Jamaica [9]). | Target goal obtained from conversations with the Jamaica MoH [5]. |
| Screening for neuropathy, and protective footwear to treat individuals with neuropathy | 7% | 37% | The baseline represents the percent of individuals with neuropathy who are treated with protective footwear (based on estimates provided by the Diabetes Association of Jamaica [9]). | Target goal obtained from conversations with the Jamaica MoH [5]. |
| **Depression** | | | | |
| Basic psychosocial treatment for mild cases | 15% | 50% | The baseline represents the percent of individuals with mild forms of depression who receive basic psychosocial treatment [5]). | Target goal obtained from conversations with the Jamaica MoH [5]). |
| Basic/intensive psychosocial treatment and anti-depressant medication for first episode moderate-severe cases | 34% | 68% | The baseline represents the percent of individuals with moderate or severe depression who receive basic or intensive psychosocial treatment [5]). | Target goal obtained from conversations with the Jamaica MoH [5]). |
| Intensive psychosocial treatment and anti-depressant medication of recurrent moderate- severe cases (on an episodic or maintenance basis) | 36% | 54.4% | The baseline represents the percent of individuals with moderate or severe depression who receive basic or intensive psychosocial treatment [5]), where individuals who experience three or more episodes of depression receive treatment on a maintenance basis. | Target goal obtained from conversations with the Jamaica MoH [5]). |
| **Anxiety** | | | | |
| Basic psychosocial treatment for mild cases | 11% | 50% | The baseline represents the percent of individuals with mild forms of anxiety who receive basic psychosocial treatment [5]). | Target goal obtained from conversations with the Jamaica MoH [5]). |
| Basic psychosocial treatment and anti-depressant medication for first episode moderate-severe cases | 20% | 52% | The baseline represents the percent of individuals with moderate or severe forms of anxiety who receive basic psychosocial treatment [5]). | Target goal obtained from conversations with the Jamaica MoH [5]). |
| Intensive psychosocial treatment and anti-depressant medication for first episode moderate-severe cases | 6% | 30% | The baseline represents the percent of individuals with moderate or severe forms of anxiety who receive intensive psychosocial treatment [5]). | Target goal obtained from conversations with the Jamaica MoH [5]). |

a Clinical interventions are scaled linearly between the baseline and target over the 15-year time frame of the investment case analysis. For policies, the investment case assumes that implementation or intensification of new policy measures cannot take place until year three of the analysis, allowing time for development and planning of new policies, and aligning with the cost structure for policies as laid out in the WHO NCD Costing Tool (see Section

# Health benefits

## Modeling

The health benefits that result from moving policies and clinical interventions from baseline to target goals are obtained using the NCD Impact Module of the inter-UN agency OneHealth Tool (OHT). The impact module contains a collection of multistate lifetables that model the extent to which the population experiences health events and the likelihood of death (see CVD example in S1 Fig). Estimates of the initial prevalence of each health state, transitions between health states (incidence), and mortality are sourced from the 2010 Global Burden of Disease database, as are disability weights for various health states associated with each disease or disorder.

The modelling and assumptions behind clinical interventions that address CVD and diabetes are detailed in Ortegón et al (2012) [10]. The modelling and assumptions behind clinical interventions that address depression and anxiety are detailed in Chisholm et al (2016) [11].

S1 Fig. Health-state transitions within the OneHealth Tool (OHT) Impact Module's CVD platform

** Individuals may transition to “death” from any health state*

## The impact of clinical interventions and policies that target NCDs

### Clinical interventions

S1 Table 2. Effect sizes of clinical interventions that address CVD, diabetes, depression, and anxiety

| **Intervention** | **Effect Size** | **Source** |
| --- | --- | --- |
| **Cardiovascular disease** | | |
| Treatment for individuals with high CVD risk (≥ 20%) | 1.05 mmol/L reduction in cholesterol  5.9mmHg reduction in systolic blood pressure | [12, 13] |
| Treatment for individuals with high blood pressure (≥ 140 mmHg), but low absolute CVD risk (< 20%) | 5.9mmHg reduction in systolic blood pressure | [12] |
| Treatment for individuals with high cholesterol (≥ 6.0 mmol/L), but low absolute CVD risk (< 20%) | 1.05 mmol/L reduction in cholesterol | [13] |
| Treat new cases of acute myocardial infarction with aspirin | 15% reduction in CVD mortality | [14] |
| Provide multidrug therapy to treat those with established ischemic heart disease and stroke | 1.05 mmol/L reduction in cholesterol  5.9mmHg reduction in systolic blood pressure | [12, 13] |
| **Diabetes** | | |
| Standard glycemic control | 75% reduction in the incidence of retinopathy | [15] |
| Intensive glycemic control | 65% reduction in the incidence of retinopathy | [15] |
| Treatment for individuals with sight-threatening retinopathy (laser photocoagulation therapy) | 80% reduction in blindness due to retinopathy | [16] |
| Treatment for individuals with neuropathy (protective footwear) | 50% reduction in lower-limb amputation due to severe neuropathy | [17] |
| **Depression** | | |
| Basic psychosocial treatment for mild cases of depression | 15% improvement in remission, 3.9% improvement in functioning | [11] |
| Basic and intensive psychosocial treatment and anti-depressant medication for moderate to severe cases of depression (first episode cases) | 21-24.5% improvement in remission, 6.1-9.7% improvement in functioning | [11] |
| Intensive psychosocial treatment and anti-depressant medication for moderate to severe cases of depression (for severe first episode cases, and moderate to severe recurrent cases) | 24.5% improvement in remission, 9.7% improvement in functioning | [11] |
| **Anxiety** | | |
| Basic psychosocial treatment for mild cases of anxiety | 36% improvement in remission, 7.4% improvement in functioning | [11] |
| Basic psychosocial treatment and anti-depressant medication for moderate to severe cases of anxiety | 36% improvement in remission, 9.2% improvement in functioning | [11] |
| Intensive psychosocial treatment and anti-depressant medication for moderate to severe cases of anxiety | 42% improvement in remission, 12% improvement in functioning | [11] |

### Policy measures

S1 Table 3. Effect sizes of policy measures that reduce demand for tobacco and alcohol

| **Policy measures** | **Effect Size  (Absolute reduction^a^ in tobacco use prevalence, or hazardous use of alcohol)** | **Source** |
| --- | --- | --- |
| Tobacco | | |
| Implement and enforce bans on smoking in all public places | 4% | [18] |
| Implement and enforce bans on tobacco advertising, promotion, and sponsorship (TAPS) | 10% | [18] |
| Increase taxes | ^b^ | - |
| Implement plain/standardized packaging of tobacco products | 0.55% | [19] |
| Institute mass media campaigns | 3.8% | [18] |
| Alcohol | | |
| Implement and enforce restrictions on alcohol advertising, promotion, and sponsorship (TAPS) | 1.2% | [20] |
| Implement and enforce restrictions on the physical availability of alcoholic beverages | 2.1% | [20] |
| Increase taxes | ^b^ | - |

a The absolute reductions displayed in S1 Table 3 represent the impact from having no policy in place to having policy in place that reflects international standards (see Target goals in S1 Table 1) and that is well enforced. Within the investment case, gradations of these effect sizes are used based on how the extent to which Jamaica already has implemented the policy at baseline.

b The impact of the tobacco and alcohol tax increases are mediated by the size of the increase in price generated by the tax increases (see Baseline and Target coverages), elasticity of consumption (-0.5 for cigarettes [21], and -0.3 for beer, -0.45 for wine, and -0.55 for liquor [4]), and other factors such as unrecorded use of alcohol.

### Baseline risk factors prevalence

| **Males** | **15-19** | **20-24** | **25-29** | **30-39** | **40-49** | **50-59** | **60-69** | **70-79** | **80+** | **Source** |
| --- | --- | --- | --- | --- | --- | --- | --- | --- | --- | --- |
| Hazardous alcohol use (%) | 2.3 | 24.5 | 20.2 | 18.8 | 20.4 | 20.4 | 17.4 | 9.4 | 9.4 | [22] |
| Tobacco (%) | 9.0 | 20.3 | 19.6 | 21.4 | 20.0 | 17.9 | 18.9 | 6.1 | 6.2 | [8] |
| Mean systolic blood pressure (mmHg) | 118.9 | 118.9 | 119.2 | 121.3 | 128.6 | 136.7 | 138.9 | 143.6 | 143.6 | [23] |
| Mean total cholesterol (mmol/L) | 4.0 | 4.0 | 4.2 | 4.3 | 4.4 | 4.5 | 4.5 | 4.7 | 4.7 | [23] |
| Mean BMI | 23.2 | 23.2 | 23.3 | 24.5 | 26.1 | 26.1 | 25.2 | 20.5 | 20.0 | [23] |
|  |  |  |  |  |  |  |  |  |  |  |
| **Females** | **15-19** | **20-24** | **25-29** | **30-39** | **40-49** | **50-59** | **60-69** | **70-79** | **80+** | **Source** |
| Hazardous alcohol use (%) | 9.8 | 9.2 | 9.2 | 7.4 | 5.3 | 4.0 | 2.8 | 0.8 | 0.8 | [22] |
| Tobacco (%) | 3.5 | 7.1 | 5.8 | 6.5 | 5.8 | 4.8 | 5.2 | 0.9 | 0.7 | [8] |
| Mean systolic blood pressure (mmHg) | 111.1 | 111.1 | 114.7 | 117.8 | 127.1 | 135.8 | 141.5 | 150.4 | 150.4 | [23] |
| Mean total cholesterol (mmol/L) | 4.3 | 4.3 | 4.4 | 4.5 | 4.7 | 4.8 | 4.8 | 5.2 | 5.2 | [23] |
| Mean BMI | 24.4 | 24.4 | 28.3 | 29.4 | 30.4 | 30.3 | 29.9 | 27.0 | 24.0 | [23] |

# The costs of policy measures and clinical interventions

## Policy measures

The financial costs to the government of implementing tobacco and alcohol policy measures—or of intensifying or enforcing existing ones—are estimated using the Excel-based WHO NCD Costing Tool [24].

The Tool contains default costs from 2008, which are sourced from the WHO CHOICE costing study. Following Shang and colleagues (2018), the Tool was updated to reflect 2016 costs by updating several parameters: the USD to local currency unit exchange rate (2016), purchasing power parity (PPP) exchange rate (2016), GDP per capita (USD, 2016), GDP per capital (PPP, 2016), population (total, and share of the population age 15+, 2016), labor force participation rate (2016), and government spending on health as a percent of total health spending (2016) [25, p. 5].

S1 Table 4. 15-year financial costs of policy measures (Discounted, 2016 currency units, millions)

| **Policy measures** | **JMD** | **USD** |
| --- | --- | --- |
| Tobacco | | |
| Program costs | 214.9 | 1.7 |
| Enforce bans on smoking in all public places | 60.9 | 0.5 |
| Implement and enforce bans on tobacco advertising, promotion, and sponsorship (TAPS) | 167.8 | 1.3 |
| Increase taxes | 250.4 | 1.9 |
| Implement plain/standardized packaging of tobacco products | 162.3 | 1.3 |
| Institute mass media campaigns | 311.6 | 3.5 |
| Alcohol | | |
| Program costs | 214.9 | 1.7 |
| Implement and enforce restrictions on alcohol advertising, promotion, and sponsorship | 144.2 | 1.1 |
| Implement and enforce restrictions on the physical availability of alcoholic beverages | 685.3 | 5.4 |
| Increase taxes | 223.4 | 1.8 |

## Clinical Interventions

To estimate the cost of scaling up clinical interventions that address CVD, diabetes, and mental health disorders, the investment case draws on resource estimates embedded in the OneHealth Tool. The Tool contains treatment assumptions for each clinical intervention, including the medicines, diagnostics, and provider time (e.g., number and length of annual outpatient visits, hospital inpatient days, therapy sessions) required to treat each patient.

In addition, the Tool contains default data on the unit cost of each medicine and diagnostic. This data is sourced from the MSH International Medical Products Price Guide [26], and is the median buyer price of the medicine or diagnostic within its dataset. Where possible, country partners replaced unit cost estimates with local data on prices of medicines or diagnostics.

The investment case uses country-specific estimates from WHO CHOICE on the cost of outpatient visits or hospital bed-days at various levels of the health system [27]. These costs include health and non-health personnel costs, capital costs, utilities, and other direct and indirect costs, but exclude medicines and diagnostic costs. All costs in the WHO CHOICE dataset are updated from 2008 to 2016 currency units using Jamaica’s local consumer price index [28].

Accounting for distribution costs to ship and distribute medicines and ensure that they are widely available, the investment case assigns a 16 percent markup on medicines and diagnostics. The parameter is the midpoint of a range specified in a USAID study that found that in more developed countries (such as Jamaica), where existing public and private sector infrastructure is in place to meet logistics needs, supply chain costs to distribute essential drugs and medicines are estimated to result in about a 12-20 percent markup on their wholesale cost [29]. This markup is added onto the cost of medicines and diagnostics.

Within the Mental Health package, supportive costs to facilitate scale up are included, including, program, training, outreach, and media. These costs are derived from the Jamaica Mental Health and Homeless Task Force report, the Ministry of Health’s Proposal for implementation of Recommendations from the Task Force on Mental Health and Homelessness, and the mhGAP Costing Tool [30, 31].

**S1** **Table 5. Per person annual treatment cost, by intervention**

| **Intervention name** | **Cost (JMD)** | **Cost (USD)** |
| --- | --- | --- |
| **Cardiovascular disease** | | |
| Screening for CVD Risk | 1,871 | 15 |
| Treatment for those with high blood pressure but low absolute risk of CVD (<20%) | 5,673 | 45 |
| Treatment for those with high cholesterol but low absolute risk of CVD (<20%) | 5,750 | 45 |
| Treatment for those with a 10-year risk of a CVD event (≥ 20%) | 7,858 | 62 |
| Treatment of cases with established IHD and Post MI | 15,580 | 123 |
| Treatment of cases with established cerebrovascular disease | 55,540 | 437 |
| Treatment of new cases of acute IHD | 105,000^a^ | 827 |
| **Diabetes** | | |
| Standard Glycemic Control (60% of diabetics receive standard glycemic control) | 27,532 | 217 |
| Intensive Glycemic Control (40% of diabetics receive intensive glycemic control) | 33,237 | 262 |
| Screening for neuropathy | 1,506 | 12 |
| Treatment for neuropathy | 20,805 | 163 |
| Screening for retinopathy | 2,000^b^ | 16 |
| Laser photocoagulation therapy to treat retinopathy | 57,500^b^ | 453 |
| Lower-limb amputation | 1,500,000^c^ | 11,900 |
| **Depression** | | |
| Basic psychosocial treatment for mild depression | 2,496 | 20 |
| Basic psychosocial treatment and anti-depressant medication for first episode moderate-severe cases | 6,466 | 51 |
| Intensive psychosocial treatment and anti-depressant medication of first-episode cases, and recurrent cases on an episodic basis | 39,906 | 314 |
| **Anxiety** | | |
| Basic psychosocial treatment for mild anxiety | 2,496 | 20 |
| Basic psychosocial treatment and medication for anxiety disorders (moderate-severe cases) | 6,466 | 51 |
| Intensive psychosocial treatment and medication for anxiety disorders (moderate-severe cases) | 39,906 | 314 |

**Note**: Unless otherwise marked with an asterisk, costs are reflective of treatment assumptions embedded in the OHT, and resource costs provided by the Jamaica National Health Fund database or OHT, and WHO CHOICE.

a Source: Fray-Aiken and colleagues (2016) [32]

b Source: Diabetes Association of Jamaica [9].

c Average of amputation costs in four Latin America and the Caribbean countries (Trinidad and Tobago, Brazil, Peru, Chile) [33-36]. Costs from the literature adjusted to 2016 JMD currency units.

# Monetizing health benefits: formulas and approach

**Section 4** describes the parameters used to value the extent to which interventions restore individual’s capacity to spend time engaged in economic activities. The economic benefits are a function of the amount of time that an individual spends engaged in productive economic activities. Interventions increase (or restore) productive time for individuals by decreasing the chance that they will die prematurely; by protecting their capacity to participate in the workforce, or; by allowing them to work more frequently or capably because of improved health. In addition, economic value is derived from individuals avoiding healthcare costs due to improved health or avoided disease/disabling events.

## Monetizing gains from avoided mortality

Mortality removes individuals from the workforce, reducing human capital and obviating the economic contributions that they would have made during the remainder of their expected life. The value of avoided premature mortality is obtained by multiplying the number of deaths avoided by labor force participation rates (63.4 percent [37]) by the expected economic contribution of each worker (valued at GDP per employed person, JMD 1.5 million) [37, 38].

## Monetizing gains from increases in the labor force

Disabling states such as blindness and lower-limb amputation, and episodes of depression or anxiety lower individuals’ likelihood of participating in the workforce.

In Jamaica, employment rates of individuals who are blind or disabled are reported to be below two percent [39, 40]. The economic gains from avoided labor force exit due to blindness are measured by subtracting the number of individuals who would be expected to be employed with a disability from the number expected to be employed in the absence of a disability, multiplied by GDP per employed person to obtain the value of labor exit avoided. Following the methodology of Chisholm and colleagues (2016), interventions to treat depression and anxiety are modeled to increase the labor force participation rate (LFPR) of individuals with mental health disorders by five percent [11].

## Monetizing gains in labor productivity

Individuals with poor health are more likely to miss days of work (absenteeism) or to work at a reduced capacity while at work (presenteeism). Labor productivity gains from avoided morbidity were measured by multiplying reductions in the incidence of diseases (or in the case of mental health disorders by the number of cases of remission from episodes of depression or anxiety), the labor force participation rate, the expected change in working days (see S1 Table 6), and GDP per employed person.

S1 Table 6: Absenteeism and Presenteeism estimates from academic literature, by disease/disease event

| **Disease/Disease event** | **Loss - # of days**^a^ | **Loss - % of working year** | **Source** |
| --- | --- | --- | --- |
| Presenteeism | | | |
| Stroke or IHD event survivor | 8.9 | 0.037 | [41] |
| Depression | 24 | 0.10 | [11, 42, 43]^b^ |
| Anxiety | 24 | 0.10 | [11, 42, 43] |
| Absenteeism | | | |
| Stroke or IHD event survivor | 2.7 | 0.011 | [44] |
| Depression | 12 | 0.05 | [11, 42, 43]^b^ |
| Anxiety | 12 | 0.05 | [11, 42, 43] |

a Assumes 240 days of work per year

b Chisholm and colleagues (2016) reviewed 440 published trials of psychological and anti-depressant medication interventions for depression and identified three trials that reported the impact of treatment on absenteeism. All three trials found that treatment reduced the number of missed working days (absenteeism) by about 1 day per month (12 days in a year). Two studies reported presenteeism separately, finding that between 1-3 working days per month (average of 2 days in a month or 24 days in a year) are lost from presenteeism.

## Estimating the value of avoided healthcare expenditures

Primary and secondary prevention efforts lead to less disease and fewer disease events, meaning that the government, households, and private insurers avoid healthcare expenditures that would otherwise have been needed for treatment.

Within the investment case, avoided healthcare expenditures are estimated for a limited set of diseases and disease events.

- Direct costs avoided by preventing strokes and IHD events
- Direct costs avoided by preventing sight-threatening retinopathy and amputations due to severe neuropathy.

The number of people who would have been treated for each disease or disease event, is multiplied by the cost of the treatment.

## The Return on Investment

The return on investment is calculated as the present value of economic benefits (sum of avoided mortality, exit from the workforce, presenteeism, absenteeism, and healthcare costs) divided by the present value of the financial costs of the interventions.

# References

1. WHO Report on the Global Tobacco Epidemic: Jamaica. World Health Organization, 2015 2015. Report No.

2. WHO REPORT ON THE GLOBAL TOBACCO EPIDEMIC, 2017. In: Organization WH, editor. online2016.

3. World Health Organization. Global strategy to reduce the harmful use of alcohol. Geneva, Switzerland: 2010.

4. Nelson JP. Meta-analysis of alcohol price and income elasticities--with corrections for publication bias. Health Economics Review. 2014;17.

5. Davis T. Conversations with the Jamaica Ministry of Health. 2017.

6. National Strategic and Action Plan for the Prevention and Control of Non-Communicable Diseases (NCDS) in Jamaica 2013 - 2018. Ministry of Health, 2013.

7. Cunningham-Myrie C, Younger-Coleman N, Tulloch-Reid M, McFarlane S, Francis D, Ferguson T, et al. Diabetes mellitus in Jamaica: sex differences in burden, risk factors, awareness, treatment and control in a developing country. Trop Med Int Health. 2013;18(11):1365-78. doi: 10.1111/tmi.12190.

8. Wilks R, Younger N, Tulloch-Reid M, McFarlane S, Francis D. Jamaica Health and Lifestyle Survey: Technical Report. Jamaica Health and Lifestyle Research Group, 2008 2008. Report No.

9. Less L. Correspondence with the Diabetes Association of Jamaica. 2017.

10. Ortegón M, Lim S, Chisholm D, Mendis S. Cost effectiveness of strategies to combat cardiovascular disease, diabetes, and tobacco use in sub-Saharan Africa and South East Asia: mathematical modelling study. BMJ. 3442012.

11. Chisholm D, Sweeny K, Sheehan P, Rasmussen B, Smit F, Cuijpers P, et al. Scaling-up treatment of depression and anxiety: a global return on investment analysis. The lancet Psychiatry. 2016;3(5):415-24. Epub 2016/04/17. doi: 10.1016/s2215-0366(16)30024-4. PubMed PMID: 27083119.

12. Law MR, Morris JK, Wald NJ. Use of blood pressure lowering drugs in the prevention of cardiovascular disease: meta-analysis of 147 randomised trials in the context of expectations from prospective epidemiological studies. Bmj. 2009;338:b1665. Epub 2009/05/21. doi: 10.1136/bmj.b1665. PubMed PMID: 19454737; PubMed Central PMCID: PMCPMC2684577.

13. Taylor F, Huffman MD, Macedo AF, Moore TH, Burke M, Davey Smith G, et al. Statins for the primary prevention of cardiovascular disease. Cochrane Database Syst Rev. 2013;(1):Cd004816. Epub 2013/02/27. doi: 10.1002/14651858.CD004816.pub5. PubMed PMID: 23440795; PubMed Central PMCID: PMCPMC6481400.

14. Collaborative meta-analysis of randomised trials of antiplatelet therapy for prevention of death, myocardial infarction, and stroke in high risk patients. Bmj. 2002;324(7329):71-86. Epub 2002/01/12. doi: 10.1136/bmj.324.7329.71. PubMed PMID: 11786451; PubMed Central PMCID: PMCPMC64503.

15. Eastman RC, Javitt JC, Herman WH, Dasbach EJ, Zbrozek AS, Dong F, et al. Model of complications of NIDDM. I. Model construction and assumptions. Diabetes Care. 1997;20(5):725-34. Epub 1997/05/01. PubMed PMID: 9135934.

16. American Diabetes Association. Diabetic Retinopathy. 2002 Contract No.: (Suppl 1).

17. Apelqvist J, Larsson J. What is the most effective way to reduce incidence of amputation in the diabetic foot? Diabetes Metab Res Rev. 2000;16 Suppl 1:S75-83. Epub 2000/10/31. PubMed PMID: 11054894.

18. Levy DT, Tam J, Kuo C, Fong GT, Chaloupka F. The Impact of Implementing Tobacco Control Policies: The 2017 Tobacco Control Policy Scorecard. J Public Health Manag Pract. 2018. Epub 2018/01/19. doi: 10.1097/phh.0000000000000780. PubMed PMID: 29346189.

19. Chipty T. Study of the Impact of the Tobacco Plain Packaging Measure on Smoking Prevalence in Australia health.gov.au2016 [4/16/2018]. Available from: <https://www.health.gov.au/internet/main/publishing.nsf/content/491CE0444F7B0A76CA257FBE00195BF3/$File/PIR%20of%20Tobacco%20Plain%20Packaging%20-%20with%20Addendum.docx>.

20. Cook WK, Bond J, Greenfield TK. Are Alcohol Policies Associated with Alcohol Consumption in Low- and Middle-Income Countries? Addiction. 2014;109(7):1081-90. doi: 10.1111/add.12571. PubMed PMID: 24716508; PubMed Central PMCID: PMC4107632.

21. Walbeek CB, J.; Booth, A.; Lewis, F. A Review of Excise Taxes in Jamaica. online: 2015.

22. Younger-Coleman N, Cumberbalch C, Campbell J, Ebanks C, Williams D, O'Meally V. National Drug Use Prevalence Survey: Technical Report. National Council on Drug Abuse, 2016 2016. Report No.

23. World Health Organization. Review of comparable estimates of selected NCD risk factors. Unpublished 2010.

24. Costing Tool – User Guide - Scaling Up Action against Noncommunicable Diseases: How Much Will It Cost? World Health Organization, 2012.

25. Shang C, Yadav A, Stoklosa M, Kontsevaya A, Lewis FB, Pana A, et al. Country-specific costs of implementing the WHO FCTC tobacco control policies and potential financing sources. PLoS One. 2018;13(10):e0204903. Epub 2018/10/04. doi: 10.1371/journal.pone.0204903. PubMed PMID: 30281668.

26. International Medical Products Price Guide online2015. Available from: <https://www.msh.org/resources/international-medical-products-price-guide>.

27. World Health Organization. Country-specific unit costs for primary and secondary health care services Online2010 [cited 2017]. Available from: <https://www.who.int/choice/country/country_specific/en/>.

28. Consumer price index. In: International Monetary Fund IFSadf, editor. The World Bank Data Catelog2017.

29. Sarley D, Allain L, Akkihal A. Estimating the Global In-Country Supply Chain Costs of Meeting the MDGs by 2015. Arlington, Va: USAID | DELIVER PROJECT, Task Order 1., 2009.

30. Task Force on Mental Health and Homelessness. Report; Mental Health and Homeless Task Force. Ministry of Health, 2017.

31. The Task Force on Mental Health and Homelessness. Proposal for implementation of recommendations from the Task Force on Mental Health and Homelessness. Ministry of Health, 2017.

32. Fray-Aiken W, Abdulkadri, McCaw-Binns. Cost of care of chronic non-communicable diseases in Jamaican patients: the role of obesity. Farmeconomia. 2016;17(2).

33. Cardenas MK, Mirelman AJ, Galvin CJ, Lazo-Porras M, Pinto M, Miranda JJ, et al. The cost of illness attributable to diabetic foot and cost-effectiveness of secondary prevention in Peru. BMC Health Serv Res. 2015;15:483. Epub 2015/10/28. doi: 10.1186/s12913-015-1141-4. PubMed PMID: 26503154; PubMed Central PMCID: PMCPMC4623251.

34. Cavanagh P, Attinger C, Abbas Z, Bal A, Rojas N, Xu ZR. Cost of treating diabetic foot ulcers in five different countries. Diabetes Metab Res Rev. 2012;28 Suppl 1:107-11. Epub 2012/02/01. doi: 10.1002/dmrr.2245. PubMed PMID: 22271734.

35. Cawich SO, Islam S, Hariharan S, Harnarayan P, Budhooram S, Ramsewak S, et al. The Economic Impact of Hospitalization for Diabetic Foot Infections in a Caribbean Nation. Perm J. 2014;18(1):e101-e4. doi: 10.7812/TPP/13-096.

36. Toscano CM, Sugita TH, Rosa MQM, Pedrosa HC, Rosa RDS, Bahia LR. Annual Direct Medical Costs of Diabetic Foot Disease in Brazil: A Cost of Illness Study. Int J Environ Res Public Health. 2018;15(1). Epub 2018/01/11. doi: 10.3390/ijerph15010089. PubMed PMID: 29316689; PubMed Central PMCID: PMCPMC5800188.

37. Statistical Institute of Jamaica. Demographic and Social statistics - Main Labour Force Indicators. Online2016.

38. Statistical Institute of Jamaica. Economic statistics - Annual GDP Statistics. Online2016.

39. Employers urged to hire more visally impaired workers. Jamaica Gleaner. 2016/10/17.

40. The World Bank. Online. 2016. [cited 2017]. Available from: <http://www.worldbank.org/en/news/feature/2016/04/18/acting-on-disability-discrimination-jamaica>.

41. Wang PS, Beck A, Berglund P, Leutzinger JA, Pronk N, Richling D, et al. Chronic medical conditions and work performance in the health and work performance questionnaire calibration surveys. J Occup Environ Med. 2003;45(12):1303-11. Epub 2003/12/11. doi: 10.1097/01.jom.0000100200.90573.df. PubMed PMID: 14665817.

42. Harvey et al. Work and depression/anxiety disorders – a systematic review of reviews. online: Beyondblue, 2012.

43. Woo JM, Kim W, Hwang TY, Frick KD, Choi BH, Seo YJ, et al. Impact of depression on work productivity and its improvement after outpatient treatment with antidepressants. Value in health : the journal of the International Society for Pharmacoeconomics and Outcomes Research. 2011;14(4):475-82. Epub 2011/06/15. doi: 10.1016/j.jval.2010.11.006. PubMed PMID: 21669372.

44. Anesetti-Rothermel A, Sambamoorthi U. Physical and mental illness burden: disability days among working adults. Popul Health Manag. 2011;14(5):223-30. Epub 2011/04/22. doi: 10.1089/pop.2010.0049. PubMed PMID: 21506731.
