# Supplementary material for: The investment case as a mechanism for addressing the NCD burden: Evaluating the NCD institutional context in Jamaica, and the return on investment of select interventions
Source: PLoS One. 2019 Oct 4;14(10):e0223412. doi: 10.1371/journal.pone.0223412 (PMC6777795; doi:10.1371/journal.pone.0223412)
Supplement: S2 Supporting Appendix — (DOCX) [file pone.0223412.s002.docx]

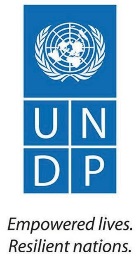


**Institutional and Context Analysis for the Prevention and Control of Non-communicable Diseases**

S2 Supporting Appendix. Institutional and Context Analysis - Methods and Background

**Introduction and purpose**

Non-communicable diseases (NCDs) – mainly cardiovascular disease, diabetes, cancer and chronic respiratory disease – are the single greatest cause of preventable illness, disability and mortality worldwide, responsible for more deaths than all other causes combined [1]. Low– and middle-income countries account for 75 percent of NCDs deaths globally, and 80 percent of premature deaths from NCDs [2]. With NCDs holding back not just health but social, economic and environmental objectives more broadly, the [2030 Agenda for Sustainable Development](https://sustainabledevelopment.un.org/post2015/transformingourworld) recognizes the fact that NCD trends and sustainable development cannot coexist. The Agenda calls for reducing premature mortality from NCDs by one-third, and strengthening implementation of the World Health Organization Framework Convention on Tobacco Control (WHO FCTC) in all countries (see Annex 2).

Meeting these ambitious targets will require more than strengthened leadership and action from the health sector. Experiences in [addressing the social determinants of NCDs](http://www.undp.org/content/undp/en/home/librarypage/hiv-aids/discussion-paper--addressing-the-social-determinants-of-noncommu.html) demonstrate that policy choices spanning across government – on finance, trade, tax, labour, agriculture and education, for example – often have a greater bearing on NCD outcomes than do healthcare sector policies per se. Significant, complementary action from other sectors and stakeholders is therefore crucial. Multisectoral action has been endorsed as cornerstone of NCD responses in the [2011 Political Declaration on the Prevention and Control of NCDs](http://www.who.int/nmh/events/un_ncd_summit2011/political_declaration_en.pdf) and numerous other high-level political decisions.

Securing support from non-health sectors for sustainable national NCD responses, while essential, presents unique governance challenges and requires a thorough understanding of political and institutional contexts. The four main behavioural risk factors that drive NCD epidemics – tobacco use, harmful use of alcohol, unhealthy diet and physical inactivity – are enmeshed with commercial and broader public-private interests that often conflict with attempts to improve public health by reducing the consumption of health-harming products. Even well-intentioned stakeholders, including non-health sectors of government, often fail to take into account how their decisions and policies may exacerbate NCDs, and do not fully recognize the social and economic costs of NCDs. Amongst these are the significant indirect costs from people who are no longer able to work, or who work less effectively, due to NCD-related death and disease. When stakeholders compare the economic benefits from an unaltered or minimally altered NCD environment solely against the public health gains of NCD prevention and control, the door opens for commercial interests to take advantage of any lax standards in managing industry-government relationships to ensure that their interests are preserved in public policy. The result is that even the most proven and cost-effective NCD prevention and control policies, such as those articulated in WHO’s Best Buys, the WHO Global NCD Action Plan 2013-2020 (WHO GAP 2013-2020) or the WHO FCTC, can be difficult to implement.

Institutional and context analyses (ICAs) – sometimes referred to as political economy analyses (PEAs) – help provide the information needed to identify and address political and other contextual challenges, ideally during the planning phase of development programmes. An institutional and context analysis seeks to define how diverse institutions in a society shape the likelihood of programmatic success [3]. According to the World Bank, PEAs help programme designers and managers “focus on power and resources, how they are distributed and contested in different country and sector contexts, and the resulting implications for development outcomes” [4]. ICAs:

- Seek to define the key institutional and governance arrangements and capacities, the political economy drivers, and entry points and risks relevant to an intervention. They also seek to evaluate priorities and potential for change;
- Can be applied at country, sector and project levels, and may consider factors such as institutional and governance arrangements, interests, incentives, historical legacies, prior experience with reforms, social trends, and how all of these factors effect or impede change; and
- Help to improve project design, increase the likelihood that human, technical and financial resources can be effectively utilized and project objectives delivered, explain the likely distributional aspects of reform efforts, and promote more thoughtful and effective multi-stakeholder engagement with client governments and other actors (S2 Fig).

**S2 Fig. How do diverse institutions in a society shape the likelihood of programme/policy success?**

In 2012, the UNDP Oslo Governance Centre published a [Guidance Note for conducting ICAs](http://www.undp.org/content/undp/en/home/librarypage/democratic-governance/oslo_governance_centre/Institutional_and_Context_Analysis_Guidance_Note.html). Here UNDP has adapted the Oslo Guidance Note to develop a Guiding ICA Framework that can be applied to the unique challenge of determining policy for NCD prevention and control. The Framework is intended for UNDP, WHO and other partners such as UNFPA and UNICEF who support national NCD costing exercises/investment case analyses, and participate in Joint Programming Missions to countries on NCDs. In 2017, UNDP updated its 2012 Guidance Note on ICAs in the context of the 2030 Agenda. The resulting Guidance Note, [Institutional and Context Analysis for the Sustainable Development Goals](http://www.2030agenda.undp.org/content/dam/2030agenda/Publications/Institutional_and_Context_Analysis_August_2017.pdf), incorporates experiences and lessons from an early NCD-specific ICA carried out in Mongolia.

**S2 Box 1. Purpose of the Tool**

This tool will help assess the political and economic dimensions of NCD policy adoption, implementation and enforcement, including how a robust return to investment analysis would affect these dimensions. The overall goal is to examine/determine the political space for implementing priority NCD-relevant interventions, and for UNDP, WHO, and other partners to best advise on the specific strategies and approaches most likely to increase that space – in other words, to conduct an ICA that is tailored to NCDs. The findings will support the development, financing and implementation of national multisectoral NCD strategies, in furtherance of the SDGs, the 2011 Political Declaration on NCDs and as stipulated in WHO GAP 2013-2020. ICAs directly complement investment cases and inform any associated advocacy and messaging. The investment cases utilize a costing model to measure the health and economic impacts/gains of various NCD-relevant interventions to generate a recommended set of cost-effective priority NCD interventions, but do not adequately take political context into account.

**Guiding ICA Framework for the prevention and control of NCDs**

**S2 Table 1 The framework**

| **Step** | **Overarching question** | **Sample considerations** |
| --- | --- | --- |
| 1. Define the scope of the problem and assess the opportunities/   challenges for responses. | *“What are the needs, opportunities and challenges for NCD-related interventions?”* | - 1. **How much and what type of policy attention do NCDs receive at national level?** Has the country stated priorities on NCDs? Are NCDs considered in national health and development planning processes/instruments? How responsive are these processes/instruments to epidemiological burdens and stated priorities on NCDs? Are there national NCD targets? If yes, how do these align with global NCD targets?   2. **What NCD-relevant policies and interventions already exist?** Is the country a Party to the WHO FCTC? If no, why not? If yes, what is the progress on implementation? Are there any laws regulating key NCD risk factors, including tobacco, alcohol and diet? What/who has been the catalyst/driver for these?   3. **What are – and have historically been – the challenges/barriers to the adoption, implementation and enforcement of NCD-related interventions?** |
| 1. Determine institutional and governance arrangements and capacities. | *“Who are the relevant actors, how do they operate, and are they capable effective, and efficient?”* | - 1. **Which institutions, sectors of government and other stakeholders including industry (collectively ‘actors’) influence the country’s NCD-related targets, plans and interventions?** What are their roles, responsibilities and capacities (defined and informal)? Is there clarity/agreement over these?   2. **What are the policy processes, structures and arrangements through which NCDs and health are governed?** What is the level/nature of interaction, coordination and cooperation among and between actors? What has driven this historically?   3. **How are NCD-related interventions understood and defined among actors?** Is there clear agreement as to what constitutes an NCD intervention? To what extent are multisectoral approaches and whole-of-society engagement recognized/valued?   4. **Which actors have generally supported NCD-related interventions, and which have generally opposed them?** What have been the common arguments for and against NCD-related interventions? Who makes them and are they successful?   5. **Relative to each other, which actors have the most/least political influence, money and resources?** How capable, effective and efficient is each actor in either advancing or impeding NCD-related interventions? |
| 1. Assess available and potential resources. | *“What current and potential mechanisms, strategies and opportunities exist for financing NCD responses?”* | - 1. **Are existing and planned NCD policies/interventions budgeted for?**   2. **Is the country receiving – or has it previously received – ODA for NCD responses?** From regional and multilateral channels, such as the development banks? If yes, what for?   3. **Have innovative domestic financing strategies been considered and/or implemented**, including taxes on health-harming products, analyzing public expenditures across sectors to ensure coherence with NCD policies/objectives, identifying high-value integrated responses, etc.? |
| 1. Identify the political economy drivers. | *“What are the political, economic and other priorities/incentives of the relevant actors – and how do these relate, broadly, to NCD-related interventions?”* | - 1. **What are the main interests and priorities (political/economic/other) of the relevant actors?** What incentives drive their core business? Are these aligned with the country’s NCD-related policy objectives? How are priorities shaped by political/election cycles?   2. **What type of exchanges/transactions do the relevant actors undertake, including with each other?**   3. **Upon what main information do actors make their decisions?** What is the political appetite for, and responsiveness to, cost-effective analysis and evidence-based arguments?   4. **Are there international influences on domestic NCD policymaking (e.g. international trade agreements/disputes, multinational corporations, etc.)?** What broader social, economic and political trends/forces are relevant to national NCD responses?   5. **Does the country consider itself a policy leader in the region?** If so, how does this affect decision making and attitudes to policy risk management? |
| 1. Propose priority actions and identify key supporters and key opposition. | *“Which cost-effective NCD-related interventions are most feasible given the political and economic context, and how are relevant actors likely to perceive them?”* | - 1. **Based on the information collected, which cost-effective NCD-related interventions are most/least likely to be supported across a critical mass of actors?** What are the pros/cons of each, and how do they advance or impede (or not affect) the interests of the most influential actors?   2. **What are the most feasible entry points for introducing these interventions in the short–, medium– and long-term?**   3. **Given interests, priorities and incentives, as well as historical legacies, which actors are most likely to support strengthened NCD responses, and which are most likely to oppose them** (i.e. which actors gain from the status quo, and which gain from a strengthened NCD response)?   4. **Which actors would bring the most traction to a strengthened NCD response, and which would be the most powerful opposition?** What specific arguments will the opposition likely make against the priority actions? |
| 1. Evaluate potential for change and identify enabling factors/   strategies. | *“How likely to be implemented are the priority actions and what factors/ strategies can expand the political space for adoption, implementation and enforcement?”* | - 1. **Are the priority NCD-related interventions likely to be implemented within the existing political space?**   2. **How can ‘allied’ actors be empowered/supported to promote the NCD-related interventions?** What other strategies and political/financial resources can improve the likelihood of success (e.g. can media, NGOs and/or civil society be engaged to counter the opposition’s potential arguments)?   3. **What is the optimal timing, tailoring, and sequencing of the priority NCD actions** (e.g. how can key windows of political opportunity be maximized, such as national planning/programme processes)?   4. **Are there other promising approaches for expanding political support for sustainable NCD responses** (e.g. can certain private sector interests and/or broader social, economic and political forces be leveraged)?   5. **How can public awareness and debate be harnessed to drive policy change or influence institutional decisions**? |

***Activities and outputs***

With UNDP and WHO Country Office support, implement UNDP’s Guiding ICA Framework for the prevention and control of NCDs (outlined in the S2 Table 1) to assess the political and economic dimensions of NCD-relevant policy adoption, implementation and enforcement. Specific activities and outputs include:

- **Conduct pre-mission desk research on the context of the participating country**, including on NCD burdens, including how NCDs and their risk factors are distributed across populations, and other relevant information (e.g. population size, development/poverty level, socio-economic profile, broader epidemiological trends). The desk review should at minimum include relevant results from the following WHO surveys: STEPS (STEPwise Approach to Surveillance), GATS (Global Adult Tobacco Survey) and GYTS (Global Youth Tobacco Survey). The pre-mission desk research should also examine media coverage, if any, of NCD burdens, trends and policy responses (or lack thereof).
- **Conduct a comprehensive landscape analysis** to determine factors such as: existing and planned NCD prevention and treatment interventions; the governance structures, agencies, civil society organizations, private sector and other key stakeholders involved in the NCD response – and the influence/views of each; potential bottlenecks to NCD-relevant interventions, for example conflicts of incentives; existing and potential financing mechanisms/resources for NCDs; and promising approaches/strategies for implementing multisectoral prevention strategies. Annex 1 presents considerations and topics for stakeholder discussions. Also useful for discussions are Annex 2, on NCDs and the SDGs, and the [multisectoral NCD policy briefs](http://www.undp.org/content/undp/en/home/librarypage/hiv-aids/what-government-ministries-need-to-know-about-non-communicable-diseases.html), developed by WHO and UNDP.
- **Document results, experiences, recommendations and lessons learned in an ICA report.** The report should outline a clear plan for increasing the political space to adopt, implement and enforce NCD-relevant interventions, including by overcoming identified blockages, mitigating conflicts of interest between public and private actors, and ensuring increased policy coherence across sectors of government. The report will be finalized in consultation with UNDP, WHO and other designated colleagues/counterparts. Recommendations from the ICA can assist in the communications strategy that ensures that an investment case is heard, understood and acted on.

**References**

1. Global Burden of Disease 2013 Mortality and Causes of Death Collaborators. 2015. “Global, regional, and national age-sex specific all-cause mortality for 240 causes of death, 1990-2013: a systematic analysis for the Global Burden of Disease Study 2013. *Lancet*, 385: 117-71.

2. WHO 2015. Factsheet on non-communicable diseases. Available at: <http://www.who.int/mediacentre/factsheets/fs355/en/>

3. UNDP, “Institutional and Context Analysis Guidance Note,” 2012, available [here](http://www.undp.org/content/undp/en/home/librarypage/democratic-governance/oslo_governance_centre/Institutional_and_Context_Analysis_Guidance_Note.html).

4. Alice Poole, Public Sector and Governance Group, World Bank, “How-To Notes: Political Economy Analysis at Sector and Project Levels,” 2011, available [here](http://www.gsdrc.org/docs/open/pe1.pdf).

5. Basu, S, et al. 2011. “Projected effects of tobacco smoking on worldwide tuberculosis control: mathematical modelling analysis.” *BMJ*, 343, doi: <http://dx.doi.org/10.1136/bmj.d5506>

6. Yen, Y-F, et al. 2014. “Smoking increases risk of recurrence after successful anti-tuberculosis treatment: a population-based study.” *The International Journal of Tuberculosis and Lung Disease,* 18(4): 492-498.

7. Putting noncommunicable diseases on the global agenda: NCD Alliance briefing paper: NCDs, Tobacco control, and the FCTC. NCD Alliance; 2011. Available at: <http://www.ncdalliance.org/sites/default/files/rfiles/NCD%20Alliance%20Briefing%20Paper%20NCDs%20%20Tobacco%20Control%20and%20the%20FCTC_0.pdf>

8. Bloom D et al. The Global Economic Burden of Noncommunicable Diseases. World Economic Forum (WEF) and Harvard School of Public Health (HSPH); 2011.

9. From Burden to “Best Buys”: reducing the Economic Impact of Non-Communicable Diseases in Low- and Middle-Income Countries. Geneva: World Economic Forum (WEF) and World Health Organization (WHO). 2011.

10. A/RES/70/1. Resolution adopted by the General Assembly on 25 September 2015. Transforming our world: the 2030 Agenda for Sustainable Development. Available at: <http://www.un.org/ga/search/view_doc.asp?symbol=A/RES/70/1&Lang=E>

11. Frank J. Chaloupka, Ayda Yurekli, and Geoffrey T. Fong, “Tobacco Taxes as a Tobacco Control Strategy,” *Tobacco Control*, 2012, 21 pp. 172-180, <http://tobaccocontrol.bmj.com/content/21/2/172.full>.

**Annex 1 – ICA interviews and topics**

The heart of the ICA is the stakeholder interviews undertaken during the comprehensive landscape analysis. Experiences to date – in Barbados, Fiji, Krygyzstan, Mongolia, Belaraus and Jamaica – demonstrate the array of potentially relevant stakeholders to interview. Which particular stakeholder groups to meet should be determined on a case-by-case basis, accounting for national circumstance, MoH input and scheduling possibilities. Who to meet within stakeholder groups must also be considered. Though not always, technical staff may offer the best chance to uncover details on ‘what is really happening’, while Permanent Secretaries/Directors may be more politically guarded. On the other hand, Permanent Secretaries/Directors offer an opportunity to influence a higher-level audience. Ultimately it makes sense for the ICA team to meet with both political and technical staff (thought not necessarily at the same time), as the ICA has a dual purpose of information extraction and advocacy.

The pros and cons of various approaches in conducting interviews must also be assessed. S2 Table 2 below offers suggested – but not prescriptive – topic areas for various stakeholders. Indeed, some conversations may proceed best when structured against a sequential set of standard questions/areas, while others may feel stifled or forced if the conversation does not flow iteratively and uninterrupted. Moreover, in some cases a larger group of interviewers/facilitators (i.e. a larger stakeholder analysis team) may be appropriate, whereas in others a larger group may be intimidating and deter information extraction. Likewise, a multi-stakeholder forum may be ideal in some circumstances, whereas in others bilateral meetings are optimal. Finally, the use of computers for notetaking should also be carefully considered. While it is critical to document information quickly and efficiently, fast and visible typing may not be welcomed by a stakeholder who is taking a perceived risk to provide/discuss sensitive information. More appropriate may be handwritten notes or quickly recording information post-interview. All of these (and still other) factors will need determination by the ICA mission team in real time.

**S2 Table 2. Potential ICA interviews and topics to raise.**

| **Stakeholder** | **Topics^a^** |
| --- | --- |
| *All* | |
| All | Purpose of IC/UNIATF; stakeholder’s current contribution to national NCD response, including collaborations with MoH + other stakeholders; opportunities to expand collaboration in light of IC findings; opportunities for NCD response integration or strengthening in specific strategies/plans; implementation of coordination/mechanisms; perception of ‘grand narratives’ in the country, e.g. what got the government elected, where is the political capital – and how NCDs tie to that. |
| *Joint meetings with the whole investment case team* | |
| MoH inception meeting | Status and directions of NCDs/national response; who are the key stakeholders within MoH/across gov & society; any sensitivities to be aware of; how and why would the MoH find an investment case useful? |
| UNCT inception meeting | UNCT engagement on NCDs (projects, partnerships, frameworks, UNDAF) and roles of agencies; opportunities for expanded support in the context of the investment case + SDGs and development partner interest. |
| NGOs (ideally a forum where many attend – then follow up bilaterally as needed) | Nature, extent and influence of contributions to national NCD response (e.g. service provision, advocacy, accountability); gaps and opportunities for expanded support in light of investment case findings; working relationship and trends with state institutions. |
| *ICA interviews* | |
| Consumer protection agency | Health-harming products – contents, addictive properties, package labelling, menu labelling, pictorial warnings, marketing (esp. to children); alternative medicines, information and legislation/regulation; nature of oversight and enforcement. |
| Federation of employers | Economic impact of NCDs; workplace health and wellness programming; (mis)notions of “job loss” and reduced economic activity from stronger NCD action; broader support to national NCD response. |
| Chamber of commerce | Economic impact of NCDs; workplace health and wellness programming; (mis)notions of “job loss” and reduced economic activity from stronger NCD action; broader support to national NCD response; industry influence in policymaking + marketing practices particularly pertaining to children; codes of conduct knowledge and adherence; what more could members do in NCD prevention and control space? |
| Transparency international (or similar NGO) | Industry influence in policymaking; policy coherence for NCDs; potential support to national NCD response/building capacities of key stakeholders (e.g. parliamentarians); codes of conduct. |
| Government accountability or anti-corruption agency | Industry intereference in policymaking; policy coherence for NCDs; potential support to national NCD response/building capacities of key stakeholders (e.g. parliamentarians); codes of conduct. |
| Public health foundation (if existent) | Contribution to NCD response, including treatment vs. health-promotion focus; costs of services; sources of funding (e.g. taxation of health-harming products/private sector partnerships). |
| Ministry of tax and duties | NCD economics. Taxation of health-harming products; other innovative strategies (e.g. fuel tax, import/export duties); mis(notions) of job loss, reduced economic activity, regressivity, illicit trade; importance of regional economic commissions, trade agreements and standards. |
| World Bank and/or other IFIs | NCDs and development; purpose of IC; past, current and pipeline loans re: NCDs; opportunities for UNIATF technical support/synergy with investment case through loan design and support. |
| Executives of private sector entities | Economic impact of NCDs; private sector and NCDs (both influence and contributions, recognizing heterogeneity); workplace wellness; opportunity to fill private sector leadership vacuum; codes of conduct; corporate social responsibility and NCDs. |
| UNDP governance/anti-corruption/poverty reduction units (as applicable) | NCD social determinants/need for multisectoral governance; policy coherence for NCDs; industry interference in policymaking; potential support to national NCD response/building capacities of key government counterparts/through current and future projects; programme gaps and niche for UNDP. |
| UNDP CO management | NCDs as a development issue requiring a multi-sectoral and multi-UN agency response; purpose and opportunity of investment case mission; ability to strengthen existing projects/directions with greater focus on NCDs; UNDAF and UNCT roles and responsibilities. |
| Parliamentary committee on health or women’s caucus | Social, economic and environment dimensions of NCDs, including inequities; deconstruct NCD myths; duty to protect vulnerable populations in addition to health-promotion; domestic resource raising opportunities; query degree of any industry influence on policy; parliamentary codes of conduct and disclosure; previous contested policy processes in public health and lessons learned. |
| Ministry of transport | Road traffic injuries, emergency response service, air pollution measurement and policy; co-benefit possibilities through NCD action, in both financing (e.g. $ to emergency response) and projects (e.g. roads with walking/bike lanes); potential for expanded support to broader NCD agenda. |
| Ministry of education | NCDs and children; NCD risk factors in or near schools; importance of empowering children to turn the tide on NCDs; school feeding programmes; vending machine policy; NCDs in home economics curricula. |
| Ministry of culture, gender, entertainment and sports | NCDs and culture (e.g. alcohol consumption, body image perceptions, associations with traditional foodstuffs/dishes, celebrity endorsements for health-harming products vs. for pro-health behaviour;) women and NCDs (e.g. caretaking roles, tobacco industry advertising, second hand smoke exposure, links between alcohol and GBV, influence on children’s behaviour); marketing/sponsorship of sport. |
| Local government | NCDs + urbanization; NCD coordination; opportunity for leadership/innovation; NCD economics; win-win possibilities (e.g. between housing and health). |
| National and local media | Advertisement of health-harming products/restrictions + regulation; opportunities for engagement (e.g. reporting IC results, profiling people living with NCDs, calling out industry interference); conversation management through media channels; traditional health-related narratives as portrayed by media. |
| Ministry of foreign affairs and foreign trade | NCD economics (as multi-sided); health-harming products, access to medicines and trade; health obligations/human rights; need and possibility for policy coherence; regional and international influences, standards. |
| Ministry of labour | Economic impact of NCDs; workplace health and wellness programming; (mis)notions of “job loss” and reduced economic activity from stronger NCD action; broader support to national NCD response. |
| Ministry of economic growth | NCD economics; mis(notions) of job loss, reduced economic activity, regressivity, illicit trade, etc; taxation of health-harming products for health and $$; other innovative strategies (e.g. fuel tax, import/export duties). |
| Ministry of Agriculture | NCD economics; tobacco and diet/sugar sweetended beverages – taxation, food and social policies, alternative livlihoods; engagement with education/schools; import and export trends; environment and NCDs. |
| a Not exhaustive | |

**Annex 2 – NCDs and the SDGs**

The [2030 Agenda for Sustainable Development](https://sustainabledevelopment.un.org/post2015/transformingourworld), under Sustainable Development Goal (SDG) 3 on health and well-being, includes two NCD-specific targets, and several others that are NCD-relevant (S2 Box 2).

**S1 Box 2. Goal 3: ‘Ensure healthy lives and promote well-being for all at all ages’**

Target 3.3 By 2030, end the epidemics of HIV, TB and malaria^^[[1]](#footnote-1)^^

**Target 3.4 By 2030, reduce by one third premature mortality from NCDs and promote mental health and well-being**

Target 3.5 Strengthen the prevention and treatment of substance abuse, including harmful use of alcohol

Target 3.6 By 2020, halve the number of global deaths and injuries from road traffic accidents

Target 3.8 Achieve universal health coverage, including financial risk protection, access to quality essential health-care services and access to safe, effective, quality and affordable essential medicines and vaccines for all

Target 3.9 By 2030, substantially reduce the number of deaths and illnesses from hazardous chemicals and air, water and soil pollution and contamination

**Target 3.a Strengthen the implementation of the World Health Organization Framework Convention on Tobacco Control in all countries, as appropriate**

Target 3.b Support the research and development of vaccines and medicines for the…NCDs that primarily affect developing countries

Moreover, in the [Addis Ababa Action Agenda of the Third International Conference on Financing for Development](http://www.un.org/esa/ffd/wp-content/uploads/2015/08/AAAA_Outcome.pdf), Member States recognized that price and tax measures on tobacco – as called for in Article 6 of the World Health Organization Framework Convention on Tobacco Control – can reduce tobacco consumption and health-care costs while generating revenue for development financing (S2 Box 3).

**S2 Box 3. Tobacco control in the Addis Ababa Action Agenda**

**Box 3. Tobacco control in the Addis Ababa Action Agenda**

Paragraph 33: “We note the enormous burden that non-communicable diseases place on developed and developing countries. These costs are particularly challenging for small island developing States. We recognize, in particular, that, as part of a comprehensive strategy of prevention and control, **price and tax measures on tobacco can be an effective and important means to reduce tobacco consumption and health-care costs, and represent a revenue stream for financing for development in many countries**.”

Paragraph 77: “Parties to the World Health Organization Framework Convention on Tobacco Control will also **strengthen implementation of the Convention in all countries, as appropriate**, and will support mechanisms to raise awareness and mobilize resources.”

Finally, given the interdependence between NCDs and other goals and targets, progress across the SDGs, for example on poverty eradication (SDG 1) and reducing inequalities within and among countries (SDG 10), can advance NCD prevention and control and vice versa (S2 Table 3).

**S2 Table 3. NCDs and the ‘non-health’ SDGs**

| **1. No poverty** | NCDs and poverty are interlinked and mutually reinforcing. The poor face disproportionate exposures to various NCD risk factors. They also have reduced access to critical prevention and treatment services, while enduring lower levels of access to education and other health messaging opportunities. Meanwhile, NCDs can expand and deepen poverty – e.g. through catastrophic out-of-pocket medical expenditures, by forcing people to exit the labour market, and/or by pushing children out of school to act as caregivers. This reinforces the importance of adequate social protection, as well as advancing towards universal health coverage. |
| --- | --- |
| **2. Zero hunger** | Tackling NCDs means addressing malnutrition in all its forms, through approaches that do not lead to an overabundance of particularly nutrient-poor calories. Promoting alternative crops to tobacco can diversify agricultural productivity and increase income for producers, while improving land and soil quality and supporting sustainable food production systems. Reducing household spending on tobacco products can unlock resources to invest in food. |
| **4. Quality education** | Fewer NCDs among families keeps children in school. Reduced exposure to tobacco, alcohol, unhealthy diet and physical inactivity improves cognitive capacities and learning outcomes. Reducing household spending on tobacco can increase investment in education. Meanwhile, schools can provide a healthy environment, and an ideal setting for health promotion. Education’s ability to reach children at a young age and *en masse* can help establish healthy behaviours early on that can remain throughout the life course. |
| **5. Gender equality** | Women account for over half of NCD deaths and face unique NCD risk exposures, for example second-hand smoke, indoor air pollution from household chores (e.g. cookstoves) and constrained physical activity opportunities (e.g. because of unsafe neighborhoods, social norms or traditional dress). Cultural norms around ideal body size can affect not just physical but also mental health. Meanwhile, tobacco use is rising rapidly amongst women and girls, largely as a result of targeted tobacco industry campaigns that associate smoking with gender empowerment and equality. NCDs are often misconstrued as being of greater import for men, resulting in gender-related policy, programme and research gaps. |
| **6. Clean Water and Sanitation** | Access to clean water is essential for not just nutrition but also physical activity (i.e. hydration). Clean water consumption can support weight management, by contributing to fullness, stoking metabolic processes, and providing an alternative to sugar-sweetened beverages. Schools and workplaces should provide access to clean water as a key element of WHO’s Healthy Settings approach. |
| **7. Affordable and clean energy** | There are win-win innovations in health and sustainable energy with relevance for NCDs. Fuel efficient stoves can increase energy efficiency and reduce indoor air pollution (while also reducing deforestation). Equipping solar panels at health centres can ensure access to affordable, reliable and modern energy services, while also allowing health clinics to maintain cold chains and remain operational and connected. |
| **8. Decent work and economic growth** | NCDs reduce productive capacities and drag economic growth. Taxation of health-harming products is a win-win synergy between the health and economic sectors (such taxes enable people to be healthier and economies more productive, while raising government revenue and reducing health care costs down the road). Supporting economic alternatives to tobacco growing would help diversify economies. Tackling NCDs would advance better and safer working conditions, for tobacco growers, including child tobacco growers, as well as for those in extraction. Workplaces offer a strong delivery platform for health messaging as well as counselling and services provision. |
| **9. Industry, innovation, and infrastructure** | Infrastructure development raises unique health risks for workers and surrounding communities alike, including increased exposure to NCD risk factors such as harmful use of alcohol. Ensuring that capital projects are health-sensitive (i.e. take steps to mitigate increased health risks) and that their benefits are accrued by all can advance health equity, inclusive economic growth and sustainable human development broadly. Meanwhile, access to information, communications technology (including mobile health and wearable technologies), and the internet are all central to health literacy. |
| **10. Reduced inequalities** | Lower-income countries face large NCD burdens, especially disproportionately high rates of NCD-related premature mortality. Within countries, various forms of disadvantage tend to be associated with NCDs, owing partly to greater exposure to the four main behavioural risk factors and environmental risks. Many industries are increasingly targeting LMICs and vulnerable populations with advertising for health-harming products. Disadvantaged populations also face difficulties accessing essential health services and information. Conversely, reduced NCD burdens confer opportunities, for example in labour or education, which can lift people out of bad conditions. |
| **11. Sustainable cities and communities** | Urbanization offers significant opportunities for addressing NCDs, but where it is inadequately managed, urbanization can also increase exposure to NCD risk factors. Sustainable cities and communities are NCD-sensitive cities and communities; they ensure access to healthy food through innovative policy measures that improve the food environment; they focus on cleaner and more efficient forms of public transportation, thereby improving air quality, promoting physical activity (e.g. more walking and cycling) and providing speedy access to health and other services; and they are designed and regulated so as to increase people’s access to safe green spaces and smoke-free places, while reducing the risk of road traffic accidents. |
| **12. Responsible consumption and production** | Unsustainable consumption and production patterns have strong links to NCDs, for example by polluting the air, water and soil as well as altering the food supply. Removing fossil fuel subsidies, or taxing fuel, can reduce environmental harms that cause NCDs, while bringing significant revenue to government. Efforts to address NCDs push transnational corporations, and support individuals, to adopt sustainable practices for the health of the planet and its people. |
| **13. Climate action** | The links between climate and health, including NCDs, are increasingly recognized. The majority of air pollution deaths are from NCDs. Extreme weather events such as heat waves increase risk for CVD and stroke. Framing climate action as a public health priority, in addition to a planetary imperative, can generate additional support to push climate change action (e.g. the Paris Agreement of December 2015). |
| **14. Life below water** | The majority of the nearly 6 trillion cigarettes smoked each year are littered; cigarettes are in fact the most commonly littered product worldwide. The filter on cigarettes is comprised of plastic ingredients that are particularly harmful to beaches and oceans. Tobacco control, then, can reduce marine pollution/toxicity for the betterment of aquatic life. The reverse is also true: any serious efforts to improve life below water would have to engage with the specific problem of cigarettes. |
| **15. Life on land** | Tobacco farming is land intensive and frequently uses large amounts of fertilizer, herbicide and pesticide. Tobacco farming also often requires a large amount of wood for flue-curing. Taken together, tobacco production disrupts the ecosystem and leads to soil and land degradation including deforestation. Biodiversity is reduced and food insecurity increased. |
| **16. Peace and justice** | Tackling NCDs requires getting the governance right, whether through national coordination mechanisms and focal points for tobacco control, and/or multisectoral committees for NCDs. Effective governance for NCD prevention and control promotes: intersectoral engagement and conflict of interest management; transparency and accountability; reduced corruption and undue interference in policy making; and addressing organized crime such as the illicit trade of tobacco products. It supports governments to make good on their commitments, not just in addressing the four main NCD risk factors but also in ensuring access to essential medicines, delivering on the promise of universal health coverage, and addressing environmental injustices such as air pollution. |
| **17. Partnerships for the goals** | Nowhere is a “New Global Partnership” needed more than in getting business, trade and health on the same page, whether for addressing NCD risk factors or ensuring access to medicines. Win-wins are possible considering that: NCDs can actually hurt business through reduced productive capacities and increased health insurance premiums; and consumer markets are fast-developing preferences for healthier products and services. Moreover, domestic capacity for tax and other revenue collection can be enhanced by intersectoral collaboration through NCD/tobacco control mechanisms as well as specific efforts to tax tobacco and other health harming products. Tobacco control efforts can fully utilize south-south and triangular cooperation and knowledge sharing, and vice versa. |

1. NCDs are interconnected with HIV, TB and malaria, in terms of shared risk behaviours (e.g. smoking), overlapping social, economic and environmental determinants (SEEDs) and biological interactions. [↑](#footnote-ref-1)
