# Supplementary figures and images for: The investment case as a mechanism for addressing the NCD burden: Evaluating the NCD institutional context in Jamaica, and the return on investment of select interventions
Source: PLoS One. 2019 Oct 4;14(10):e0223412. doi: 10.1371/journal.pone.0223412 (PMC6777795; doi:10.1371/journal.pone.0223412)

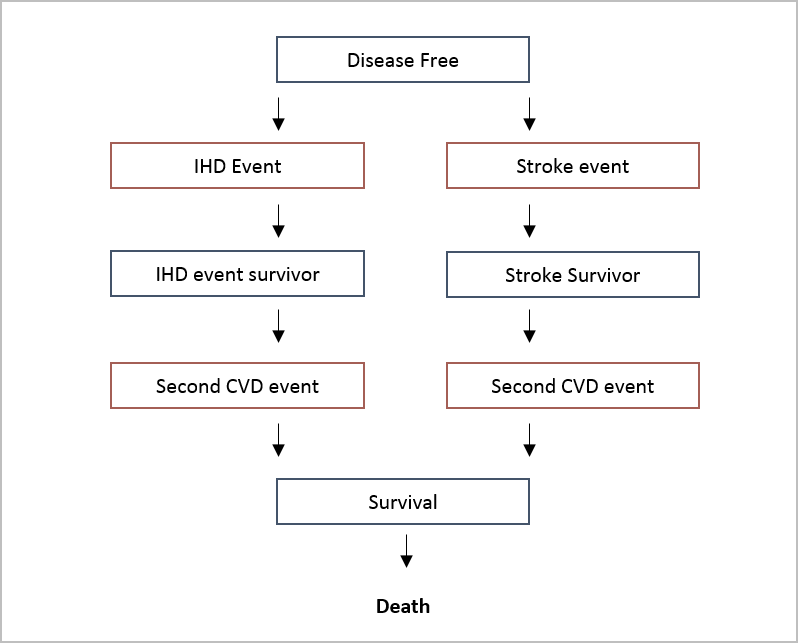

Supplement: S1 Fig — (TIF) [file pone.0223412.s004.tif]

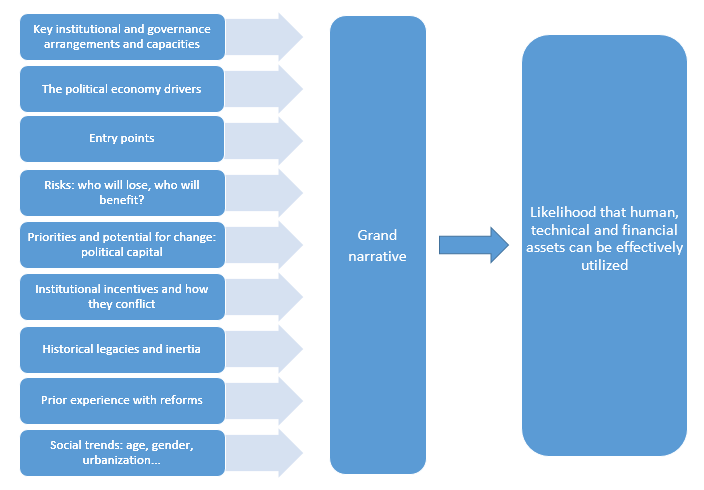

Supplement: S2 Fig — (TIF) [file pone.0223412.s005.tif]
